# Effects of geometric individualisation of a human spine model on load sharing: neuro-musculoskeletal simulation reveals significant differences in ligament and muscle contribution

**DOI:** 10.1007/s10237-022-01673-3

**Published:** 2023-01-05

**Authors:** Laura Meszaros-Beller, Maria Hammer, Julia M. Riede, Peter Pivonka, J. Paige Little, Syn Schmitt

**Affiliations:** 1 School of Mechanical, Medical and Process Engineering, Queensland University of Technology, Brisbane, Australia; 2 Institute for Modelling and Simulation of Biomechanical Systems, University of Stuttgart, Stuttgart, Germany; 3 Stuttgart Center for Simulation Science (SC SimTech), University of Stuttgart, Stuttgart, Germany

**Keywords:** Spine biomechanics, Musculoskeletal modelling, Forward-dynamics, Subject-specific, Load sharing

## Abstract

In spine research, two possibilities to generate models exist: generic (population-based) models representing the average human and subject-specific representations of individuals. Despite the increasing interest in subject specificity, individualisation of spine models remains challenging. Neuro-musculoskeletal (NMS) models enable the analysis and prediction of dynamic motions by incorporating active muscles attaching to bones that are connected using articulating joints under the assumption of rigid body dynamics. In this study, we used forward-dynamic simulations to compare a generic NMS multibody model of the thoracolumbar spine including fully articulated vertebrae, detailed musculature, passive ligaments and linear intervertebral disc (IVD) models with an individualised model to assess the contribution of individual biological structures. Individualisation was achieved by integrating skeletal geometry from computed tomography and custom-selected muscle and ligament paths. Both models underwent a gravitational settling process and a forward flexion-to-extension movement. The model-specific load distribution in an equilibrated upright position and local stiffness in the L4/5 functional spinal unit (FSU) is compared. Load sharing between occurring internal forces generated by individual biological structures and their contribution to the FSU stiffness was computed. The main finding of our simulations is an apparent shift in load sharing with individualisation from an equally distributed element contribution of IVD, ligaments and muscles in the generic spine model to a predominant muscle contribution in the individualised model depending on the analysed spine level.

**Supplementary Information:**

The online version contains supplementary material available at 10.1007/s10237-022-01673-3.

## Introduction

Research on human spine mechanics touches on the quest to understand mechanical loads exposed on and in the human body during daily life, labour and leisure activities. Especially the effects on single body parts, i.e., wear and tear of discs, joint cartilage, ligaments and muscles, are of major scientific interest. Besides experimental studies searching for mechanical (Stokes and Iatridis 2004; Smith et al. 2011; Neidlinger-Wilke et al. 2014) and genetic (Munir et al. 2018) causes of spine degeneration, a vast amount of mechanics-based simulation models have been developed to date, for example, models of single elements, such as the intervertebral disc (IVD) (e.g., Ehlers et al. 2008; Karajan et al. 2013), spinal ligaments (Zarei et al. 2018; Damm et al. 2020), facet joints (Mengoni 2021) or for their coherent representation as a functional spinal unit (FSU) (Lee et al. 2017).

Nevertheless, biological systems are not exact copies of each other. Humans differ in size, mass, strength, body composition and other more mechanically related aspects, e.g., leisure activities such as running, that can alter IVD characteristics (Belavý et al. 2017). It is therefore relevant to understand, whether and to which extent the observed differences affect the internal mechanics and to build a mechanical model accordingly. This attempt is called subject-specific modelling. It covers many aspects, e.g. simulating adolescent scoliosis surgery (Little and Adam 2011; Little et al. 2013), IVD bulge (Mengoni et al. 2021), cell death in human IVDs (Malandrino et al. 2015) and material sensitivity of lumbar IVDs (Fagan et al. 2002). Still, individualising models to an appropriate level of detail matching the respective research questions, remains a major challenge.

One challenge is the level of individualisation needed to account for relevant features. In recent years, an increasing number of musculoskeletal multibody (MB) spine models have been developed focusing on the detailed modelling of the lumbar (Christophy et al. 2012; Rupp et al. 2015; Mörl et al. 2020), thoracolumbar (Bruno et al. 2015, 2017) anatomy and musculature, and the simulation of sophisticated activities, such as various lifting (Beaucage-Gauvreau et al. 2019; Molinaro et al. 2020) or sporting tasks (Raabe and Chaudhari 2016; Cazzola et al. 2017; Silvestros et al. 2019, 2022). In a recent study by Guo et al. (2021), a full spine model was introduced demonstrating the sensitivity of spinal loading predictions to intra-abdominal pressure (IAP). These models assume generic geometries, some of which were scaled in their height or dimensions to fit a specific subject or group of the population - a start level of individualisation. Further individualisation includes adjustments with respect to muscle parameters that may vary between females (Bruno et al. 2017), males or athletes (Cazzola et al. 2017; Silvestros et al. 2022) or personalising the spinal curvature (Bruno et al. 2017). An important step towards personalised spine models was recently made in Overbergh et al. (2020) developing a framework for the inclusion of pathologic geometries derived from computed tomography (CT) scans into the kinematic chain of the model presented in Bruno et al. (2015). However, muscles have been considered symmetric with respect to the sagittal plane and passive joint stiffness was not taken into account. Despite the impressive level of individualisation achieved to date, a full subject-specific spine model, that is, a fully articulated spine with six degrees of freedom (DOF) joints at all spinal levels, driven by subject-specific muscles in a dynamic movement while restricted by subject-specific ligaments, is still not yet available.

Another challenge is the validation of human spine models. Typically, spine models are validated using literature data in so far it is available. Large experimental datasets exist in form of representative statistical means of subjects for various spine parts. For example, regarding IVD pressure, very important datasets are those of Nachemson (1975), Nachemson (1981), Wilke et al. (1999) and Wilke et al. (2001), for a recent review refer to Newell et al. (2017). For experimental data of facet joints refer to Mengoni (2021). Comprehensive validation data including the segment’s anatomy, vertebral kinematics, passive and active tissue forces and torques for individual subjects in motion, however, are still very rare. Recently, a population-specific cervical spine model was used to predict joint displacements in which rigorously validated stiffness and damping parameters of an in vitro, specimen-specific cervical model where integrated (Silvestros et al. 2019). The results prove subject-specific modelling combined with proper validation methods to be more suitable towards a better understanding of spine mechanics. In Mörl et al. (2020), experimentally determined passive spine characteristics were used to validate a generic human spine model. It was found that current ligament parameters render the spine still rather stiff.

We support the hypothesis that individualisation of the model geometry has a significant influence on internal load sharing. In this contribution, we strive to quantify the relative difference in loads at functional spinal unit (FSU) level. To answer this question, we performed forward-dynamic neuro-musculoskeletal (NMS) MB simulations and compared the results of two different human spine models, one of which is based on a generic geometry and the other one is individualised to a subject-specific dataset, by analysing their internal loads, load-sharing and joint stiffness for a forward flexion movement generated using the same motor control approach. As an additional result, we also report on the influence of different muscle co-contraction levels on load sharing. Lastly, we point out that the generic model together with the simulator is published as an open-source dataset.

## Methods

**Fig. 1:**
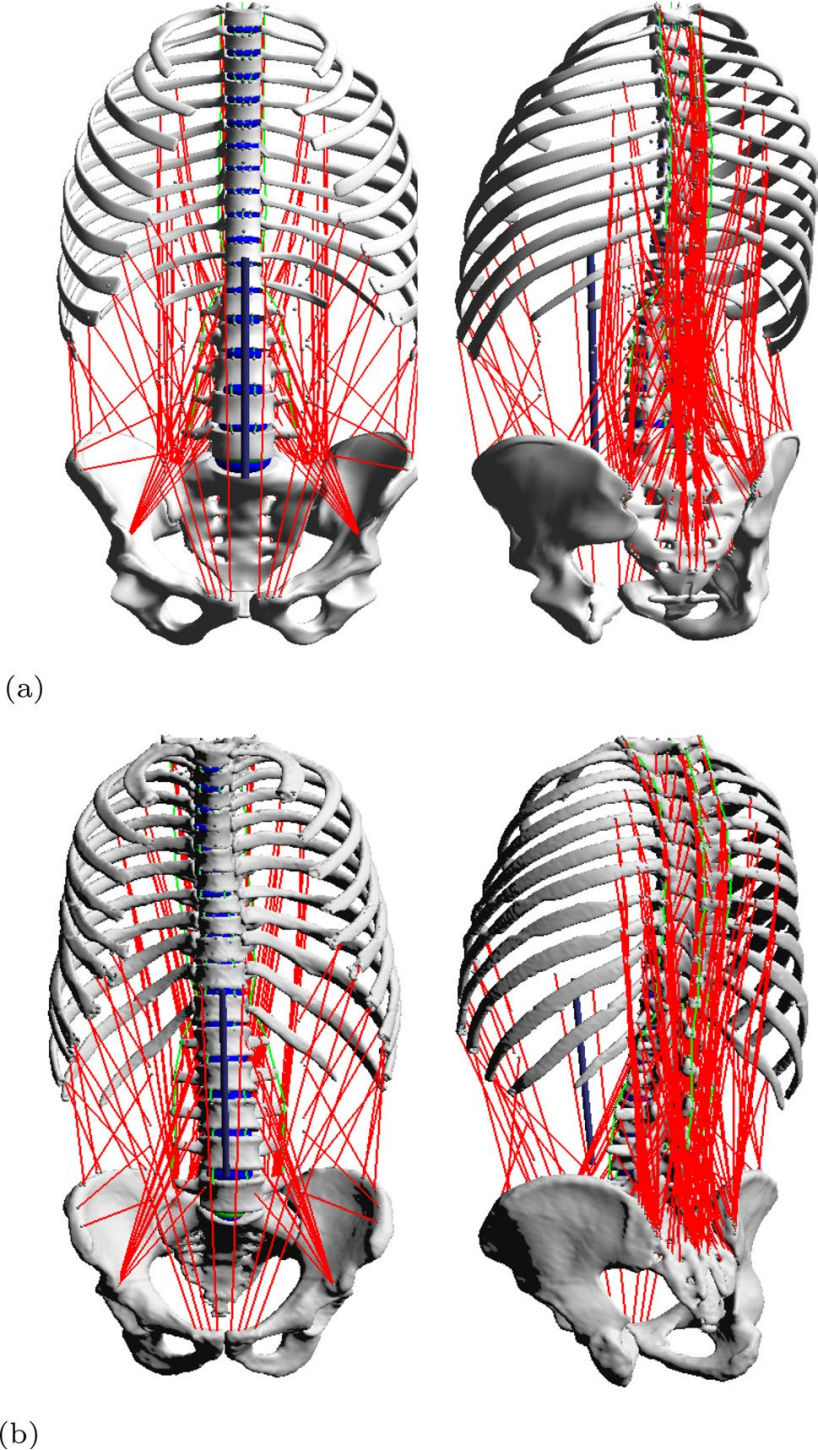
**a** The generic baseline and **b** individualised model of the thoracolumbar spine each visualised in the frontal (left) and posterolateral view (right). Both are fully articulated between T1 and S1 and incorporate individually selected muscle attachments, intervertebral disc IVDs and ligaments

The following section contains first the description of the modelling process for the generic spine model (Sect. 2.1) including modelling of the geometry and joint definition, modelling of the intervertebral discs (IVD), modelling of the muscle-tendon units and modelling of the ligaments. We used a modified version of an existing generic NMS spine model (Mörl et al. 2020) that was validated in a lying posture. In this work, we extended the previously published generic spine model to the thoracic spine region. Thereby, we particularly reviewed soft tissue stiffness parameters, muscle and ligament modelling and their line of actions by including a muscle routing algorithm. This new whole spine model serves as population-based, generic baseline model. It is available including all necessary definitions as open-source model (see ’code availability’). Second, the individualisation of the developed generic baseline model towards building the subject-specific whole spine model is described in Sect. 2.2. We individualised the baseline model with respect to its geometry (i.e., bone geometry and positions, muscle and ligament attachment points and their respective length-dependent parameters) to match the subject-specific dataset of the Visible Human Male (VHM) project (Spitzer et al. 1996). Both models (Fig. 1) consist of the same structure, i.e., same number of (1) rigid bodies representing the spinal anatomy, (2) IVDs, (3) ligaments and (4) trunk muscles. Third, the simulation part is described in Sect. 2.3 including the simulation of the settling procedure (steady-state simulation) and the forward flexion-to-extension movement. In forward dynamic simulations a flexion-to-extension movement was generated by muscle stimulation using (5) the same motor control approach allowing for a purely dynamic interaction of all elements without prescribing any kinematics. Additionally, the simulation to check controller and spine model sensitivity to variations in muscle co-contraction is also described in this section. To ensure comparability of the model outputs, internal loads were evaluated at the same lumbar flexion angle.

### Generic baseline model

#### Geometric properties and joint definition

The geometry of our generic baseline model is fully symmetric with respect to the sagittal plane of the global reference frame, and the reader is referred to Sect. 1 in Appendix for a detailed description of coordinate systems in our spine models. All vertebrae from the first thoracic (T1) to the sacrum (S1) were linked to their adjacent vertebrae by six degrees of freedom (DOF) joints allowing for relative translational and rotational movements at all spinal levels. The pelvis was rigidly connected to S1. Additionally, a thin cylindrical body anterior of the spinal column representing the linea alba (mass $=0.1\,\textrm{g}$) served as attachment site for abdominal muscles and was rigidly connected to the lowest thoracic vertebra (T12). Ribs were fixed to their corresponding vertebral body (VB) and only considered by their mass. This joint-body configuration leads to a total of 20 rigid bodies and 102 degrees of freedom (DOF)s. Analogue to our previously published model (Mörl et al. 2020; Rupp et al. 2015), individual VB locations and orientations were derived from Kitazaki and Griffin (1997) (see Appendix 2). Pelvic and thoracolumbar spine dimensions and inertia were computed for a 50th percentile male (height: $1.78\,\textrm{m}$, weight: $81.5\,\textrm{kg}$) based on the NASA anthropometric dataset (Laubach et al. 1978).

Anatomical landmarks of ligaments and muscles were selected on graphic primitives from CT data (Anatomium (TM), 21st Century Solutions Ltd/Gibraltar) to ensure physiological lines of action. Using a custom-developed graphical user interface, the VB’s centre of volume (COV) was identified for each vertebral primitive with the $z-$axis perpendicular to the primitives’ upper endplate and aligned with the local reference frame of the respective body in the kinematic chain. Furthermore, the position of the pelvis primitive (including the sacrum) was visually adjusted to the given sacrum body position and the lowest lumbar vertebra (L5) graphic primitive.

In line with the full articulation of the spinal column, the upper thorax weight was partitioned among 17 individual body segments and assigned to every spinal level between T1 and L5. The mass and relative anterior offset of the centroid of each trunk slice was derived from cadaveric studies (Pearsall et al. 1996) and defined as a point mass rigidly connected to the centre of mass (COM) of its respective VB. Further, these point masses were scaled to a total of $29.4~\textrm{kg}$ to comply with the upper and middle trunk weight of a 50th percentile male (Laubach et al. 1978) representing 36% of the desired total body weight which is in good agreement with data from Winter (2009).

**Table 1:** Linear stiffness parameters for rotational and translational movement of intervertebral disc IVDs

Stiffness parameters	Thoracic	Lumbar	Lumbo-sacral
Anterior shear ($\mathrm {N/m}$)	100,000	109,000	473,000
Lateral shear ($\mathrm {N/m}$)	110,000	130,000	523,000
Compression ($\mathrm {N/m}$)	1240,000	800,000	2420,000
Lateral bending ($\mathrm {Nm/rad}$)	172.5	200*	377
Flexion ($\mathrm {Nm/rad}$)	152	70	575
Axial rotation ($\mathrm {Nm/rad}$)	149	250	832

Parameters for the thoracic spine region were extracted from Panjabi et al. (1976), lumbar and lumbo-sacral parameters were derived from Schultz et al. (1979), Berkson et al. (1979) and Gardner-Morse and Stokes (2004), respectively. Note that the lumbar stiffness in lateral bending was increased (marked with an asterisk) to compensate for the left–right asymmetry in the individualised model

#### Modelling of the IVD

Human intervertebral joints include IVDs restricting the six-dimensional relative movements of adjacent vertebrae. In multibody (MB) simulations, they are typically included as stiffness matrices, so-called bushing elements, mimicking the force–displacement characteristics determined in experiments. We set the bushing reference frame equal to the joint reference frame and applied all IVD torques and forces at the intervertebral joint position with opposite signs onto both adjacent VBs. The components of the rotation vector were used as rotational displacements to calculate the linear bushing torques. Afterwards, these torques were transformed back into the Cartesian reference frame of the joint to account for energy conservation as in Christophy et al. (2013) and Senan and O’Reilly (2009).

Diagonal parameters for the stiffness matrices of the thoracic (T1-T12) and lumbar region (T12-L5) and the lumbo-sacral joint (L5/S1) were derived from experimental findings (Panjabi et al. 1976; Schultz et al. 1979; Berkson et al. 1979; Gardner-Morse and Stokes 2004), summarised in Table 1. We focused on stiffness parameters that encapsulate the mechanical response of an individual healthy IVD solely in all thoracolumbar levels for which we used the same literature references as in Huynh et al. (2010). Secant values for flexion and compression were averaged over both movement directions in alignment to the assumption of left–right symmetry. Values for axial and backward extension were neglected as found irrelevant for the current study. Off-diagonal values were generally set to zero; hence, coupled joint motions were neglected in the presented model. Due to the special ‘wedged’ shape of the L5/S1 IVD with occurring high shear forces and considering the absence of ligaments at this spine level in our model (see Sect. 2.1.4), we decided to use the lumped functional spinal unit (FSU) model from Gardner-Morse and Stokes (2004) at the L5/S1 motion segment.

Note the stiffness in lateral bending was increased for all lumbar IVDs from $93~\mathrm {Nm/rad}$ to $200~\mathrm {Nm/rad}$ in all simulations for both models. This was necessary in order to compensate for a slight lateral instability of the individualised model due to its left–right asymmetry and, however, had no effect on the mirror-symmetric generic spine simulations presented in this work.

Energy dissipation was included as a damping force contribution to the total IVD force

1 $$\begin{aligned} F_{i,\text {IVD},\text {damp}}= d_{\text {IVD},\text {damp}}\cdot \frac{\textrm{d}D_i}{\textrm{d}t} \quad , \end{aligned}$$

with $D_i$ denoting the translational ($i=1,2,3$) and rotational ($i=4,5,6$) displacements in the intervertebral joint, but effectively neglected by setting a uniform damping factor of $d_{\text {IVD},\text {damp}}=0.01~\mathrm {Ns/m}$ for all IVD force components and $d_{\text {IVD},\text {damp}}=0.01~\mathrm {Nms/rad}$ for IVD torque components.

Besides bushing characteristics, we considered the effect of intrinsic pressure in the nucleus pulposus. In addition to the mainly axial loading on the IVDs due to the gravitational load of the trunk (Hall 2015), intra-discal pressure (IDP) also is correlated with compressive loading at the intervertebral joints (Nachemson and Elfström 1970; Sato et al. 1999; Takahashi et al. 2006). Hence, we applied a constant offset force to every bushing element along the local longitudinal axis that was estimated from the weight of cumulated segment and VB masses located proximally to the respective intervertebral joint.

#### Muscle modelling

A total of 294 muscles were implemented in the generic baseline model. Selected muscles comprised those already implemented in our previously published model (Mörl et al. 2020) including major muscle groups for the lumbar spine, i.e. rectus abdominis (RA), external oblique (EO), internal oblique (IO), psoas major (PM), erector spinae (ES), intertransversarii mediales (IT), multifidus (MF). Due to the full articulation of the model presented in the current study, existing muscles, such as single and laminar threads of the MF, were extended to the thoracic region and additional muscles were implemented. In particular, the ES muscle group is modelled by all four lumbar and thoracic muscles, i.e. *longissimus thoracis pars lumborum*, *longissimus thoracis pars thoracis*, *iliocostalis lumborum pars lumborum* and *iliocostalis lumborum pars thoracis*. Similarly, we have implemented the *spinalis thoracis*, *semispinalis thoracis* and *interspinalis* that we will further refer to as the spinalis (SP) muscle group. A summary of all implemented muscles can be found in Tables 2 and 3 in the Supplementary data for the abdominals and back muscles, respectively.

Muscles were modelled as piecewise straight-line elements between insertion/origin and up to two intermediate deflection points using the via-ellipse method that allows a muscle thread to move within defined elliptic areas (Hammer et al. 2019). Moreover, we distinguish between soft tissue properties of tendons connecting to bones and the contractile muscle fibres; thus, we define a muscle to encapsulate the entire muscle tendon unit (MTU). A modified four-element Hill-type muscle model with an improved performance during high-frequency oscillations and eccentric contractions (Günther et al. 2007; Haeufle et al. 2014) was used to calculate the contraction dynamics of the MTU. Herein, in addition to the typical three Hill-type elements (contractile element (CE) and parallel elastic element (PEE) representing the active and passive properties of the muscle fibre and serial elastic element (SEE) representing the passive elastic tendon), a fourth element, the serial damping element (SDE), is included describing the energy dissipation in the tendon. The contraction initiated by a neuronal stimulus is governed by the activation dynamics based on Hatze (1977) and was implemented according to the newest achievements of Rockenfeller et al. (2015) and Rockenfeller and Günther (2018), respectively.

In contrast to the generic parameters of the activation and contraction dynamics, that are identical for all MTUs and were taken from Mörl et al. (2020) (see Supplementary data, Table 1), the three muscle-specific parameters maximum isometric force $F_{\text {max}}$, optimal fibre length $l_{\text {CE,opt}}$ and the tendon slack length $l_{\text {SEE,0}}$ are assigned to every MTU individually. The former was estimated from reported values for the physiological cross-sectional area (PCSA) from the literature (Christophy et al. 2012; Stokes and Gardner-Morse 1999; Bayoglu et al. 2017; Brolin et al. 2005; Delp et al. 2001) as follows:

2 $$\begin{aligned} F_{\text {max}} = \sigma \cdot \text {PCSA} \end{aligned}$$

Analogue to Mörl et al. (2020), we imply a consistent specific maximum isometric stress $\sigma ~=~23~\mathrm {N/cm^2}$ to all muscles. The characteristic lengths of the MTU ($l_{\text {CE,opt}}$, $l_{\text {SEE,0}}$) were calculated from the ratio of the muscle fibre length to the MTU length $m_{\text {ratio}}$ that was taken from experimental findings (Christophy et al. 2012; Bayoglu et al. 2017; Delp et al. 2001) or estimated from anatomical textbooks (Cramer and Darby 2005). Assuming that the trunk muscles are in their optimal isometric position during the initial posture with a total muscle length $l_{\text {MTU}}$, the characteristic muscle lengths were computed as follows:

3 $$\begin{aligned} l_{\text {CE,opt}}= & {} m_{\text {ratio}}\cdot l_{\text {MTU}} \end{aligned}$$

4 $$\begin{aligned} l_{\text {SEE,0}}= & {} l_{\text {MTU}}-l_{\text {CE,opt}} \end{aligned}$$

By doing so, muscle-specific lengths were scaled to the geometry of the generic baseline model. Muscle-specific parameters for all implemented muscles can be found in Table 2 and Table 3 in the Supplementary data.

#### Ligament modelling

Six ligaments were implemented in the baseline spine model, i.e. anterior longitudinal ligament (ALL), posterior longitudinal ligament (PLL), ligamentum flavum (LF), supraspinous ligament (SSL), intratransverse ligament (ITV) and capsular ligament (CAP), as stabilising passive straight-line elements represented by 12 threads per spinal level resulting in 192 ligament structures between T1/2 and L4/5 with the CAP and ITV implemented on both anatomical sides.

Our ligaments underlie a parametrised model of the ligaments’ stiffness curve (Günther et al. 2007) that requires a set of two characteristic points *A* ($(l_A, F_A)$ at nonlinear to linear transition) and *B* ($(l_B,F_B)$ state right before failure). The nonlinear force–displacement characteristics of all ligaments were determined by scaling force–strain data derived from the literature to the individual rest length $l_{\text {LIG,0}}$ of each ligament thread obtained from the model’s initial position. We directly used averaged thoracolumbar force–strain data from Chazal et al. (1985) for the required points *A* and *B* for the ALL, PLL, SSL and ITV ligament. In case of the LF and CAP ligaments, an optimisation was performed in order to find the optimal regression curve, i.e. the characteristic points *A* and *B*, to initial literature data from Nolte et al. (1990). We thereby have chosen the softest strain–force relation for each of the ligaments from these two datasets. The reader is referred to Appendix 4 for more details. Note we did not model the interspinal ligament ISL separately as to be irregular in humans (Johnson and Zhang 2002) and as Chazal et al. (1985) provides already combined data for SSL/ISL. Force–strain values for all ligaments used in the model with their respective literature reference indicated can be found in Table 5 in Appendix.

According to Mörl et al. (2020), the characteristic forces ($F_A,F_B$) were divided by three, as found too high otherwise, and the ligament damping factor was increased to $d_{\text {LIG,damp}}=3~\text {s/m}$ to fit experimental findings from Panjabi et al. (1998) and Hukins et al. (1990). Additionally, the resulting force values were divided by the number of parallel threads of each ligament.

Starting thoracolumbar average values for ligament prestrain $\varepsilon _{\text {LIG,init}}$ were taken from the literature: 9% for ALL (Tkaczuk 1968), 13% for PLL (Tkaczuk 1968), 11% for LF (Nachemson and Evans 1968), 9% for SSL (Robertson et al. 2013) and 5% for CAP (Gacek et al. 2022). As there was no literature available for the prestrain of the ITV, no prestrain was considered.

Tensile stress in facet joints produced in a forward flexion movement was modelled by the incorporation of the CAP ligament. In contrast to other ligaments whose origin and insertion are defined by specific landmarks, CAP ligaments were defined by selecting the mid-point of the superior facet surface of each vertebra primitive. Considering the CAP ligament enveloping a facet to be represented by a single thread perpendicular to the facet orientation, we derived the corresponding inferior facet of the superjacent vertebra from the selected superior facet for a given orientation and constant distance of 8 mm to comply with anatomical dimensions of the facet. Orientation of the CAP was defined according to the facet joint orientation in Hall (2015). As we did not simulate an extension movement, high-pressure facet contact was neglected in this study.

### Individualisation of the developed generic baseline model

For the purpose of this study we developed a partly automated framework that allows us to create subject-specific MB models based on anatomical landmarks. Subject-specific thoracolumbar anatomy, ligament attachment sites and surfaces for facet contacts were acquired from the publicly available CT dataset for the VHM using an established custom-developed modelling framework by selecting specific landmarks on the superior and inferior endplates of the VBs and the vertebral processes (Little and Adam 2015). Further, a series of user-selected landmarks defined subject-specific muscle attachments and deflection points at the thoracolumbar spine, sacrum and pelvic anatomy. Selected muscle attachments matched those already implemented in the generic baseline model. Subject-specific CT-derived segmental torso weights for each vertebral level were determined using a custom-developed software (MATLAB R2017b, The Mathworks, Natick, MA, USA) to automatically calculate the volume and centroid of the transverse CT slice and rigidly connected to the respective vertebra. A generic density of $1.04 ~\mathrm {g/cm^{3}}$ was prescribed to every greyscale pixel intensity to convert the segmental volumes into slice masses corresponding to every vertebral level (Little and Adam 2015). The determined trunk slice masses for the VHM sum up to a total of $33.8~\textrm{kg}$, which correlates well with findings from Vette et al. (2011).

In order to use the obtained subject-specific dataset (including skeletal anatomy, segment masses, muscle and ligament attachments) as an anthropometric setup, we developed a functional code that allows us to automatically build subject-specific MB models. The custom selected geometric data provided in a global reference frame include superior and inferior endplate locations and orientations, which were used to compute the global VB and joint coordinates as the average of corresponding and adjacent VB endplates, respectively, see Appendix 3. Hereinafter, the resulting global coordinates were transformed, together with muscle and ligament attachments, into local representations of individual VBs in the kinematic chain. Knowing the relative position and orientation of the VBs, joints and attachment points, the kinematic chain of our individualised model is fully defined. In accordance with our generic baseline model, the joint-body configuration of the individualised model was maintained and the linea alba defined as a thin cylinder body rigidly connected to the T12 vertebra. Anterior offset was derived from the geometric mean of pre-selected attachment points for abdominal muscles inserting to the left and right linea semilunaris. Afterwards, the spinal column was tilted forward by $2^\circ$ to meet our criterion of sagittal balance, see Appendix 1. We refer to this CT-derived geometry as the initial position of our individualised model. In the process of collecting individual anatomical data, facet joint articulating surfaces and the rest length of the CAP ligament $l_{\text {CAP,0}}$ were defined with a distance of $\mathrm {0.8~mm}$ (Little and Adam 2015). To comply with the generic baseline model, $l_{\text {CAP,0}}$ was elongated to a total of $8~\textrm{mm}$ by shifting corresponding attachment points uniformly along their distance vector.

Note, the same IVD bushing parameters (Table 1), ligament force–strain characteristics (Table 5) and muscle parameters (Supplementary data, Table 1 and  2) were used as in the generic baseline model. The muscle and ligament lengths ($l_{\text {CE,opt}}$, $l_{\text {SEE,0}}$, $l_{\text {LIG,0}}$) were, however, scaled to the individual geometry in initial position.

**Fig. 2:**
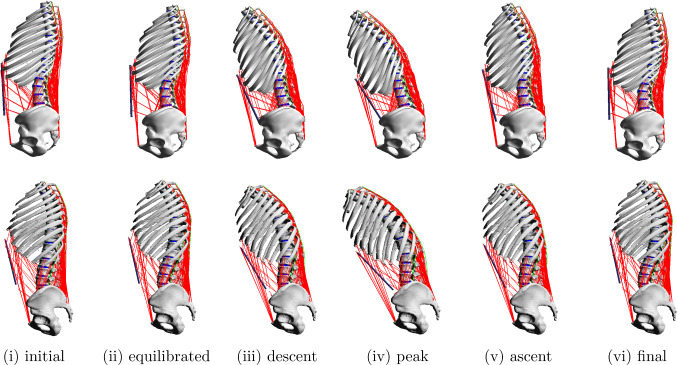
Neuromuscular-driven forward-dynamic simulations. Spine movement prediction for the generic baseline (top) and the individualised model (bottom) in the sagittal plane. Steady-state simulation (i), (ii): Starting in their respective initial positions, the models were subjected to a gravitational settling process that allowed all sub-structures to equilibrate into a loaded steady state. Forward flexion-to-extension movement (ii)–(vi): Starting in their respective equilibrated positions, the models were initiated to perform a purely muscle-driven forward flexion (descent), hold the flexed position in order to lift themselves back into upright position (ascent and final)

### Simulation

The developed models were independently used to perform a full cycle of a forward flexion-to-extension movement in a neuro-muscular driven forward-dynamic simulation starting and ending in an upright position with the pelvis spatially constrained. The movement was generated in a purely muscle-driven approach without prescribing any kinematics. The simulation was split into two separate simulations, (i) the steady-state simulation allowing the models to settle under gravity and (ii) the actual forward flexion-to-extension movement.

In both models, a bio-inspired equilibrium point control approach was used, in which muscle fibre lengths corresponding to a certain posture are considered as equilibrium point. Its control structure is based on a hybrid control scheme comprising a feed-forward open-loop stimulus representing high-level stimulation centres (central nervous system) and a sensory feedback closed-loop signal representing low-level muscle control (monosynaptic reflex):

5 $$\begin{aligned} u= & {} \frac{1}{2}\left( u_{\text {open}}+ u_{\text {closed}}\right) \nonumber \\{} & {} = \frac{1}{2}\left( u_{\text {open}}+ \kappa \cdot \frac{l_{\text {CE}}-\lambda }{l_{\text {CE,opt}}}\right) \end{aligned}$$

with *u* restricted to range between 0 (no muscle stimulation) and 1 (full muscle stimulation). The weight factors between $u_{\text {open}}$ and $u_{\text {closed}}$ were set to 0.5. The gain factor of the closed-loop signal was set to a constant of ${\kappa }=2.0$.

The direct signal from higher centres $u_{\text {open}}$ can be seen as a way to modify the co-contraction level by changing the ratio of $u_{\text {open}}$ between agonistic and antagonistic muscles. It was optimised using trial-and-error to achieve a stable equilibrated position satisfying the criterion for sagittal balance according to its definition in Appendix 1 and was assigned a constant effective level of $u_{\text {open}}^{\text {abd}}=0.02$ for the abdominal and $u_{\text {open}}^{\text {back}}=0.04$ for the back muscles, respectively, for all simulations. Note in Sect. 2.3.3 we varied $u_{\text {open}}$ for the generic baseline model to account for different co-contraction levels and its effect on load sharing.

The closed-loop signal regulates the muscle stimulation $u_{\text {closed}}$ of muscle fibres towards reaching desired muscle fibre lengths $\lambda _i$ (Eq. 5) depending on the difference between current and desired fibre length (Bayer et al. 2017; Stollenmaier et al. 2020). Thereby, the actual muscle fibre lengths $l_{\text {CE}}$ represent a specific joint angle configuration $\varphi _{\text {joint}}$, also called target position. If the target position is met, $u_{\text {closed}}$ equals zero for $\lambda _i = l_{\text {CE,i}}$. Thus, a movement can be generated by adjusting $\lambda _i$ for all muscles in the system which can be considered as a signal from the higher control level to generate motion using the low level neural structure.

#### Steady-state simulation

In the first set of simulations, both models, starting in their respective initial positions, were subjected to a gravitational settling process defined in upright position ($\varphi _{\text {spine}} = 0^\circ$) that allowed them to dynamically equilibrate into a steady state meeting the criterion for sagittal balance (Appendix 1).

At this stage, the models’ biological sub-structures (i.e. IVDs, ligaments, muscles) are found in a prestrained, loaded state in upright posture, which we will further refer to as the ‘equilibrated position’ of the models. From this equilibrated position, the reference lumbar angle $\varphi _{\text {lum,ref}}$ was derived (see Appendix 1).

All system variables describing the models’ equilibrated position, i.e. joint orientations, the bodies’ angular velocity and acceleration and the muscles’ current activity and characteristic lengths $l_{\text {CE}}$, were saved in order to provide the starting condition for the second set of simulations of a forward flexion-to-extension movement.

#### Forward flexion-to-extension movement

In the second set of simulations, the models were initiated from their respective equilibrated position in upright posture ($\varphi _{\text {spine}} = 0^\circ$) to perform a forward flexion ($\varphi _{\text {spine}} = -20^\circ$) in the sagittal plane followed by a counter movement to retrieve the upright posture ($\varphi _{\text {spine}} = 0^\circ$). The flexion movement was assessed by the change in lumbar lordosis angle $\Delta \varphi _{\text {lum}}$ with respect to the equilibrated lumbar angle $\varphi _{\text {lum,ref}}$ ($\varphi _{\text {spine}} = 0^\circ$).

According to Eq. 5, to generate the necessary muscle stimulation signals *u* for the desired motion, $u_{\text {open}}$ and $\lambda$ have to be set. The flexion movement was discretised by a sequence of pre-defined target positions $\Lambda _k$ (k: number of target positions) in a 2$^\circ$-increment in $\Delta \varphi _{\text {lum}}$ with respect to $\varphi _{\text {lum,ref}}$, each representing the desired individual muscle fibre lengths $\lambda$ of all muscles in the respective joint angle configuration $\varphi$.

Using these target positions $\Lambda _k$, the forward bending was achieved in an event-based control, i.e. by switching to the next state $\Lambda _{m} = \Lambda _{m-1}+2^\circ$ (m: m-th target position, $0\le m \le k$) when the entry condition $\Delta \varphi _{\text {lum}}\ge a\cdot \Lambda _{m-1}/2^\circ$ is met. To accelerate the initial phase of the forward flexion, we set a starting offset of 6$^\circ$. Once the desired peak flexion angle of $\Delta \varphi _{\text {lum,peak}}=-20^\circ$ was reached, the corresponding target position $\Lambda _{m,\text {{max}}}$ was held for $2\,s$ before the counter movement was initiated and target positions decremented by $\Lambda _{m}=\Lambda _{m-1}-2^\circ$, whenever $\Delta \varphi _{\text {lum}}\le b\cdot \Lambda _{m-1}/2^\circ +0.1$ was met until the model was back in its upright posture. Here again, the backward motion was initiated with -6$^\circ$ offset.

In all, we found one control policy for the forward flexion simulations to the desired $\Delta \varphi _{\text {lum,peak}}$ for both spine models. To obtain a smooth lumbar angle and to avoid overshooting only the controller parameters *a* and *b* were optimised for the two spine models, separately (generic: $a=1.68$, $b=1.93$; subject-specific: $a=1.76$, $b=1.95$).

#### Model sensitivity to muscle co-contraction

To evaluate the model sensitivity to different muscle co-contraction levels, we reran the simulations for the generic baseline model by choosing one lower and one higher level of muscle co-contraction for $u_{\text {open}}$. The lower co-contraction level of $u_{\text {open}}^{\text {abd}}= 0.01$ and $u_{\text {open}}^{\text {back}}= 0.03$ corresponded to the minimally required muscle activation for the model to be capable of free upright stance. This lower activation model recorded a decrease of $-6.2\%$ in local FSU stiffness (see Table 3). The higher co-contraction level was, hence, set to $u_{\text {open}}^{\text {abd}}= 0.03$ and $u_{\text {open}}^{\text {back}}= 0.05$ to be within similar bounds from the original muscle co-contraction. Please refer to Appendix 8 for the predicted muscle stimulations in each co-contraction condition. Note, depicted co-contraction values, for example, 2–6 represent $u_{open}=2\%/6\%$, which are effectively $1\%/3\%$ or 0.01/0.03 of *u* (see Sect. 2.3 for further details).

## Results

Using the developed generic baseline and individualised model, two neuromuscular-driven forward-dynamic simulations were performed. Firstly, an equilibrated steady state in upright position was found (Fig. 2 (i), (ii)). As simulation results, the model-specific thoracolumbar load distribution in the equilibrated position is shown and the local functional spinal unit (FSU) stiffness at L4/5 evaluated.

Secondly, initiated from the respective equilibrated state both models were promoted to undergo an approximately $-20^\circ$ forward flexion-to-extension movement (Fig. 2 (ii)–(vi)). Tracking the change in lumbar angle $\Delta \varphi _{\text {lum}}$ enabled the comparison of internal loading conditions. Exemplary for level L4/5, the load sharing between occurring internal forces generated by individual biological structures (IVDs, ligaments, muscles) and their individual contribution to the FSU stiffness is compared. Note, the display of simulation results on load sharing for the remaining lumbar joint levels can be found in Section 3 and 4 of the Supplementary data. Resulting abdominal and back muscle forces, ligament forces and strains and IVD forces and torques for the produced flexion movement are visualised separately in Appendix. The reader is referred to Appendix 5, 6 and 7 for more details.

As an additional result, the effect of different muscle activation levels on internal loading was analysed for the generic baseline model.

### Comparison of the equilibrated positions

The gravitational settling process in the steady-state simulation increased the literature-derived and CT-derived initial spinal curvatures of the generic baseline and individualised model, respectively (see Appendix 1 for the definition of spinal angles). An increase of $\Delta \varphi _{\text {lum}}^\text {generic}=2.6^\circ$ and $\Delta \varphi _{\text {tho}}^\text {generic}=4.1^\circ$ was observed in the generic baseline model and $\Delta \varphi _{\text {lum}}^\text {subject}=4.5^\circ$ and $\Delta \varphi _{\text {tho}}^\text {subject}=6.5^\circ$ in the individualised model (Table 2).

**Fig. 3:**
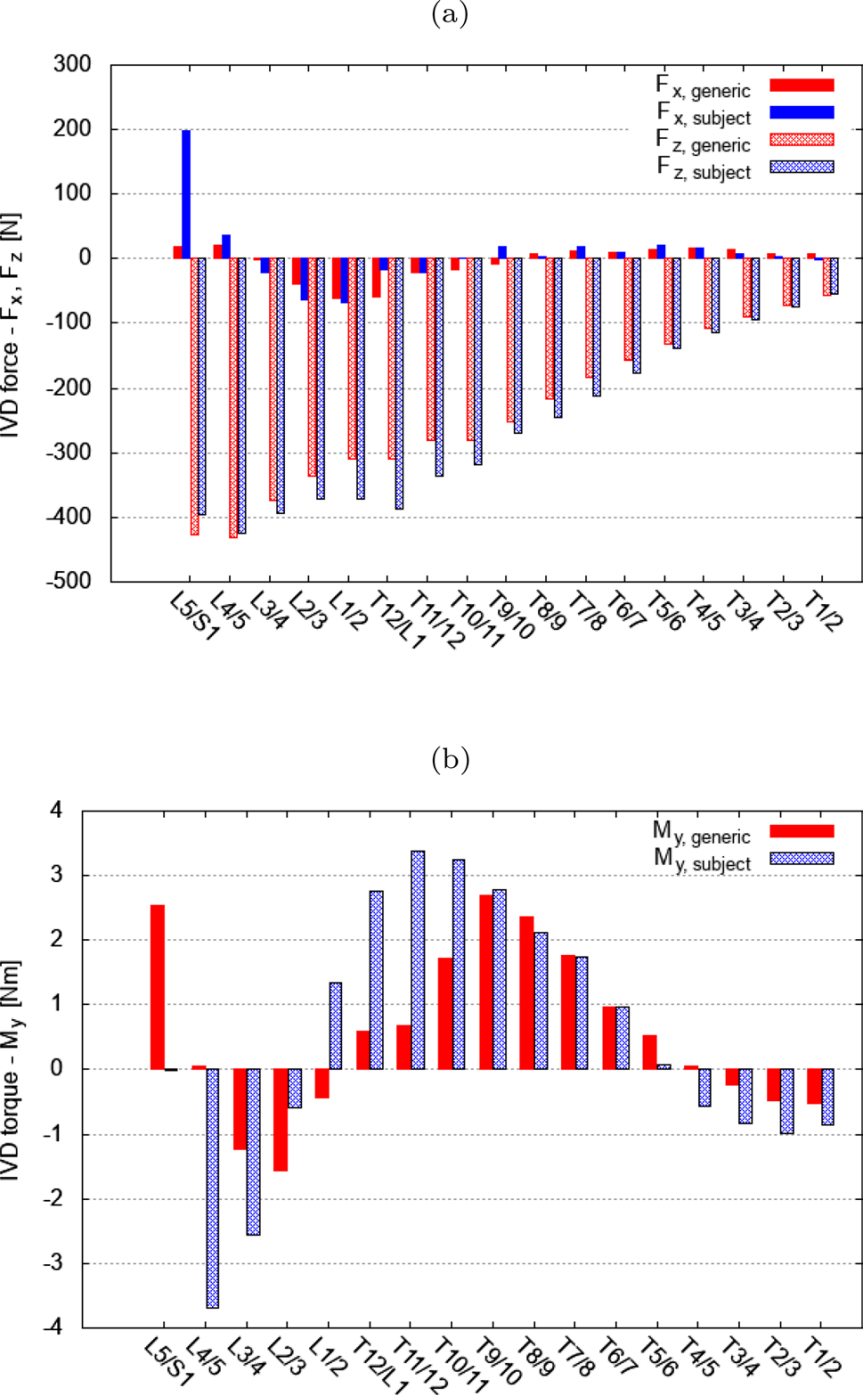
Comparison of the equilibrated positions for the generic baseline (red bars) and the individualised model (blue bars) with respect to **a** the load distribution in local longitudinal ($F_z$) and anterior–posterior direction ($F_x$), and **b** the torque in the sagittal plane $M_y$

For the display of internal loading conditions in the models’ equilibrated state, we compared the compressive IVD force $F_z$ and the IVD torque $M_y$ in Fig. 3 acting in the generic baseline (red bars) and individualised model (blue bars). Figure 3a shows a similar trend in force distribution in both models: compressive force $F_z$ increases caudally, while moderate (negative) posterior shear was recorded in the thoracolumbar transition with up to −69 N and decreased caudally and cranially except for a sharp (positive) peak of 198.5 N at level L5/S1 in the individualised model. In sum, compressive loading was 9.2% higher in the individualised model than in the generic baseline model. Maximum compressive loads were computed at level L4/5 for both models with $F_{z,\text {max}}^{\text {generic}}=-431.8~\textrm{N}$ for the generic model and $F_{z,\text {max}}^{\text {subject}}=-424.8~\textrm{N}$ for the individualised model. It is well known that the largest gravitational load acts on the lowest intervertebral joint L5/S1. This load distributes among the shear and compressive force component which is given in the local joint coordinate frame. Thus, for large rotation angles of IVDs in the sagittal plane, the compressive force can be significantly lower than the gravitational force; instead, the IVD needs to resist larger shear forces. We found the pelvis (including S1) in the CT dataset of the VHM to be significantly inclined anteriorly which implies that IVD L5/S1 is likely to experience high shear forces.

Variations between the models were found in the distribution of rotational loading acting on individual intervertebral disc IVDs as this largely depends on the manifestation of spinal curvatures. In both models anterior (positive) torque $M_{y}^{+}$ was higher in the mid-thoracic to lower thoracic spine, which is in good agreement with the larger thoracic kyphosis in this spine region. Higher posterior (negative) torque $M_{y}^{-}$ was found in the lumbar spine, going along with the typical lumbar lordosis. In the individualised model, rotational loading was 55% higher in sum than in the generic baseline model with the highest torque occurring at level T11/12 and L4/5 ($M_{y,\text {max}}^{+,\text {subject}}=3.4~\textrm{Nm}$, $M_{y,\text {max}}^{-,\text {subject}}=-3.7~\textrm{Nm}$), respectively. Peak torques in the generic baseline model occurred at level T9/10 and L2/3 ($M_{y,\text {max}}^{+,\text {generic}}=2.7~\textrm{Nm}$, $M_{y,\text {max}}^{-,\text {generic}}=-1.6~\textrm{Nm}$).

An important biomechanical factor for model comparison is the functional joint stiffness *k*. In the equilibrated position, we evaluated the local FSU stiffness $k_{\text {local,equ}}$ as the slope of the net joint torque $M_\text {y}$ for small deviations in the individual joint angle $\Delta \varphi _{L4/5}$. The local FSU stiffness in the equilibrated, upright position was $k_{local,equ}^{\text {generic}}=5.7~\text {Nm}/^\circ$ for the generic baseline and $k_{\text {local,equ}}^{\text {subject}}=5.8~\text {Nm}/^\circ$ for the individualised model under full gravitational load.

**Table 2:** Characteristic spinal angles

	Initial	Equ.	Peak	Final
*Generic*				
$\varphi _{\text {lum}}~(^\circ )$	22.5	25.05	3.92	25.15
$\varphi _{\text {tho}}~(^\circ )$	22.3	26.36	36.0	26.35
*Individualised*				
$\varphi _{\text {lum}}~(^\circ )$	16.7	21.15	− 1.02	21.54
$\varphi _{\text {tho}}~(^\circ )$	30.8	37.26	45.48	37.23

Absolute values for the characteristic curvatures of the lumbar lordosis ($\varphi _{\text {lum}}$) and thoracic kyphosis ($\varphi _{\text {tho}}$) angle for the initial, equilibrated (equ.), peak and final position of the simulated flexion-to-extension movement. In both models, $\varphi _{\text {lum}}$ and $\varphi _{\text {tho}}$ increased during the gravitational settling process. During forward flexion $\varphi _{\text {lum}}$ decreased while $\varphi _{\text {tho}}$ increased, however, start (equ.) and final values were similar

### Lumbar spine kinematics

**Fig. 4:**
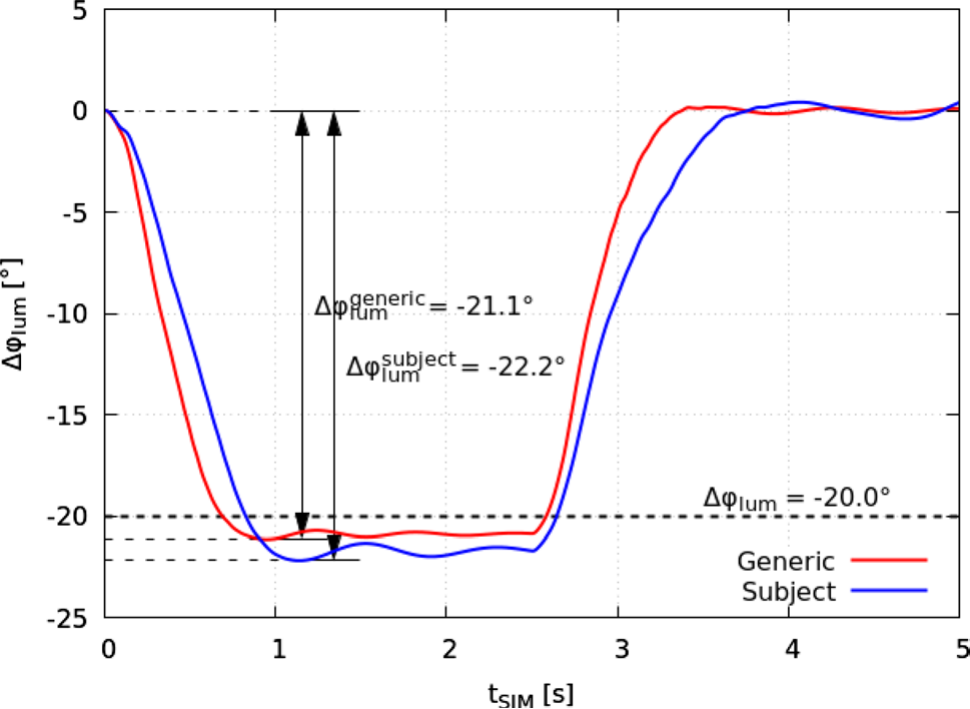
Change in lumbar lordosis angle $\Delta \varphi _{\text {lum}}$ for the generic baseline (red) and the individualised spine model (blue) for a forward flexion movement starting and ending in the equilibrated reference position. Internal loads were evaluated at $\Delta \varphi _{\text {lum}}=-20.0^\circ$ as indicated (dashed line)

The characteristic curvature of the lumbar spinal region ($\varphi _{\text {lum}}$) is subject to change during motion. Confirmed by both, the generic baseline and individualised model, the lumbar lordosis angle decreases with spinal flexion (Table 2). In Fig. 4, the change in lumbar angle $\Delta \varphi _{\text {lum}}$ is depicted for both models as a function of the simulation time $t_{\text {sim}}$. Starting in their respective equilibrated position at $t_{\text {sim}}=0\,s$, the lumbar angle decreased by $\Delta \varphi _{\text {lum}}^{\text {generic}} = -21.1^\circ$ in the generic baseline and by $\Delta \varphi _{\text {lum}}^{\text {subject}} = -22.2^\circ$ in the individualised model, respectively. The flexed position was held for $\Delta t=2\,s$; then, the spine models were triggered to return into upright position. Muscle forces to the model kinematics in Fig. 4 are depicted in Appendix 7 (Fig. 12) and show an increasing support of back muscles associated with flexion, while forward bending had no significant impact on the abdominals. For the display of results on internal loads we will use $\Delta \varphi _{\text {lum}}$ or the respective change in individual joint angle, where more appropriate, as reference.

### Model-specific structural resolution of internal forces

Increasing flexion results in an increase in active and passive muscle forces whose line of action is mostly parallel to the spinal column. In agreement with our previous findings in Mörl et al. (2020), the IVDs’ axial compression increases as a result of growing muscle contraction along with tensioning of posterior ligaments.

**Fig. 5:**
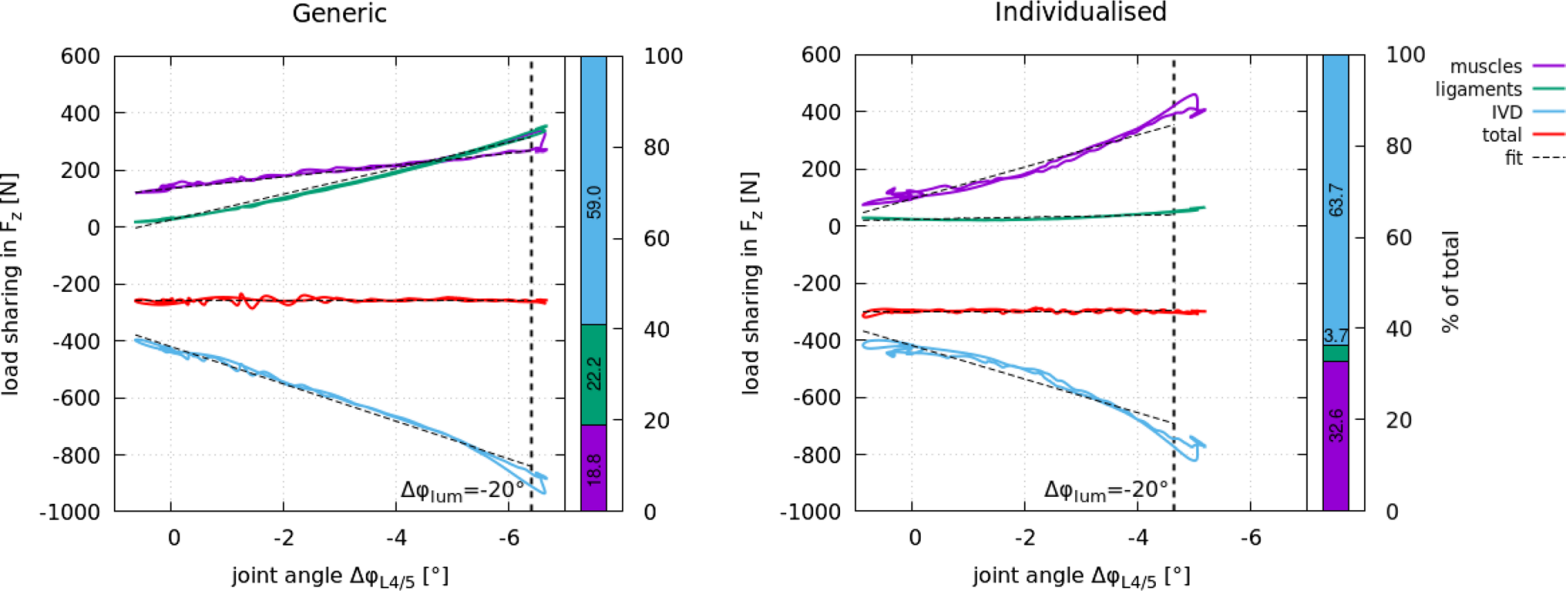
Net contributions (sums) of all anatomical structures, i.e. muscles (purple), ligaments (green) and IVDs (blue), to the net joint force ‘total’ (red) $z-$component at level L4/5 computed for the generic (left) and individualised spine model (right). Forces are defined with respect to the joints’ subjacent vertebra: compressive IVD forces are negative, while the muscles’ and ligaments’ pulling forces are positive. The dashed lines indicate the linear fit through the graphs between the minimum and maximum L4/5 joint angle for the flexion movement until $\Delta \varphi _{\text {lum}}=-20.0^\circ$ (vertical dashed line) was reached. The absolute value of the linear fit at $\Delta \varphi _{\text {lum}}=-20.0^\circ$ was used to evaluate the load sharing between anatomical structures as their relative contribution to the net joint force $F_z$ (stacked bar chart) for which the colour coding mentioned above was maintained. Each structure’s percentage contribution is stated in the corresponding bar

Figure 5 shows the net contributions of all sub-structures (muscles, ligaments and IVDs) to the net joint force along the craniocaudal axis (local $z-$axis) at level L4/5 as a function of $\Delta \varphi _{L4/5}$ for the generic baseline (left) and individualised model (right). Note, the structural resolution of internal forces for all individual lumbar joint levels can be found in Section 3 of the Supplementary data. The sum of all sub-structural forces is the net joint force (‘total’: red line), which is negative at all times during the forward flexion movement. This implies that the superior vertebra L4 applies a compressive force in caudal direction onto the subjacent vertebra L5. The increasing IVDs’ axial compression is partly counteracted by the muscles’ and ligaments’ (positive) pulling force in cranial direction. Subsequently, the net joint force stays nearly constant during spine flexion.

At level L4/5 (Fig. 5), IVD contribution to the load sharing in $F_z$ was similar between the models with 59.0% for the generic baseline and 63.7% for the individualised model at $\Delta \varphi _{\text {lum}}=-20.0^\circ$ (vertical dashed line). However, from a similar load sharing at $\Delta \varphi _{\text {lum}} \approx 0^\circ$ (equilibrated position), the ligament contribution increased more in the generic baseline model, outpacing the muscles, than in the individualised model where the muscle contribution has shown a greater increase. Subsequently, at level L4/5 ligaments contributed 6-times more to the total compressive force in the generic baseline model than in the individualised model by bearing 22.2% of the load in $F_z$. This is also reflected in Fig. 10 in Appendix where the sum of posterior ligament forces at L4/5 was significantly higher in the generic model than in the individualised model.

Consequently, at L4/5 the contribution of passive structures (IVDs + ligaments) to the net joint force decreased −14% with individualisation. However, taking all lumbar spinal levels into account (Supplementary data, Section 3) the contribution of passive structures was comparable between the models. Especially in the upper lumbar levels similar load sharing was observed with a decreasing ligament contribution caudally in the individualised model.

**Fig. 6:**
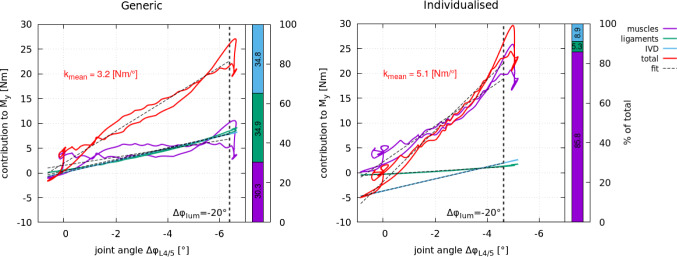
Net contributions (sums) of all anatomical structures, i.e. muscles (purple), ligaments (green) and intervertebral disc IVDs (blue), to the net joint torque ‘total’ (red) $y-$component at level L4/5 computed for the generic (left) and individualised spine model (right). The dashed lines indicate the linear fit through the graphs between the minimum and maximum L4/5 joint angle for the flexion movement until $\Delta \varphi _{\text {lum}}=-20.0^\circ$ (vertical dashed line) was reached. The absolute value of the linear fit at $\Delta \varphi _{\text {lum}}=-20.0^\circ$ was used to evaluate the load sharing between anatomical structures as their relative contribution to the net joint torque $M_y$ (stacked bar chart) for which the colour coding mentioned above was maintained. Each structure’s percentage contribution is stated in the corresponding bar. The slope of the linear fit to the sum of all net contributions (red line) was used to derive the mean FSU stiffness $k_{\text {mean}}$ indicated. The local FSU stiffness $k_{\text {local,peak}}$ in the static flexed posture was $k_{\text {local,peak}}^{\text {generic}}=8.0~\text {Nm}/^\circ$ for the generic baseline model and $k_{\text {local,peak}}^{\text {subject}}=10.9~\text {Nm}/^\circ$ for the individualised model

### Model-specific evaluation of the FSU stiffness

With spinal flexion, the contribution of all active and passive structures to the net joint torque increases. Thereby, the spine becomes stiffer with increasing flexion. The mean L4/5 FSU stiffness $k_{\text {mean}}$ can be derived as the slope of the net joint torque $M_y$ with respect to the change in joint angle $\Delta \varphi _{L4/5}$ over the course of a flexion movement. Similarly, the local FSU stiffness $k_{\text {local}}$ can be derived for small deviations in static postures.

Figure 6 shows the net contributions of all sub-structures (muscles, ligaments and IVDs) to the net joint torque $M_y$ (‘total’: red line) at level L4/5 as a function of $\Delta \varphi _{L4/5}$ for the generic baseline (left) and individualised model (right). Note, the net contributions to the net joint torque $M_y$ for all individual lumbar joint levels can be found in Section 4 of the Supplementary data. The model-specific mean FSU stiffness $k_{\text {mean}}$ was derived as the slope of the ‘total’ net joint torque using the first and last value pair of the respective linear fit (dashed line). Similarly, the model-specific local FSU stiffness $k_{\text {local}}$ was derived in the static flexed position, i.e. between 1 s and 2 s of $t_{\text {sim}}$.

At level L4/5 (Fig. 6), a different load distribution was observed between the models. In the generic baseline model, passive structures combined (IVDs + ligaments) contributed most to the net joint torque $M_y$ with 69.7%, while in the individualised model passive structures together contributed 14.2%. The passive contribution in the individualised model was counteracted by the muscles which contributed 85.8% in the individualised model. Moreover, the mean FSU stiffness $k_{\text {mean}}$ was by a factor of 1.6 higher in the individualised model than in the generic baseline model which can be attributed to the higher muscle contribution in the individualised model.

Consequently, at L4/5 the contribution of passive structures (IVDs + ligaments) to the net joint torque decreased -56% with individualisation. When taking all lumbar spinal levels into account (Supplementary data, Section 4), the generic baseline model has shown an average 30%-30%-40% load-sharing among all lumbar levels as contribution to the net joint torque by IVDs, ligaments and muscles, respectively. In the individualised model, one can observe that both IVDs and ligaments contributed increasingly less caudally resulting in a change of loading conditions in the lower lumbar spine with the highest load borne by the muscles. On average, IVDs, ligaments and muscles contributed with 20%-20%-60% to the net joint torque.

### Effect of different muscle activation levels on internal loads

Simulations as described in Sect. 2.3 were rerun for the additional activation models and compared to the ‘original’ activation model. The lower activation model included a 1% and 3% activation, while the higher activation model included a 3% and 5% activation of abdominals and back muscles, respectively. Evaluation was conducted with respect to $\varphi _{\text {lum}}$ in the equilibrated state and the load sharing in the flexion movement. Moreover, the activation-specific mean and local FSU stiffnesses ($k_{\text {mean}}$, $k_{\text {local}}$) were derived as described previously. Results are shown in Table 3.

The lower activation model had a −6.3% decline in local L4/5 FSU stiffness in the static equilibrated state, while the higher activation model, on the other hand, has shown a 5.6% increase in local L4/5 FSU stiffness in this position with respect to the ‘original’ activation model. Regarding the equilibrated state, there was a $1.1^\circ$ difference in the equilibrated lumbar lordosis angle between the lower and higher activation model. When holding the flexed position at $\Delta \varphi _{\text {lum,peak}}\approx 21^\circ$, an equal increase and decrease of $\pm 2.6\%$ in local L4/5 FSU stiffness were recorded with respect to the ‘original’ activation model.

Varying muscle activation has had a greater impact on the load sharing in $F_z$ than on the contribution to $M_y$. In all models IVD contribution was similar. In the higher activation model, however, muscles contributed $+3\%$ more to $F_z$ than in the ‘original’ activation model and $+5.7\%$ more than in the lower activation model. Ligaments contributed $-2.5\%$ and $-4.7\%$ less in the higher activation model than in the ‘original’ activation model and lower activation model, respectively. With respect to load sharing in $M_y$, there was a $+1.8\%$ increase in muscle contribution and a $-2.4\%$ decrease in ligament contribution in the higher activation model with respect to the lower activation model.

**Table 3:** Influence of different muscle activation levels on the generic baseline model at level L4/5

	Lower	Original	Higher
Abdominal/back muscle activation	1/3%	2/4%	3/5%
$\varphi _{\text {lum,equ}}$ ($^\circ$)	24.6	25.1	25.6
$k_{\text {local,equ}}$ ($\text {Nm}/^\circ$) at $\varphi _{\text {lum,equ}}$	5.4 (− 6.3%)	5.7	6.1 (+5.6%)
$\Delta \varphi _{\text {lum,peak}}$ ($^\circ$)	− 21.0	-21.1	− 21.4
$k_{\text {local,peak}}$ ($\text {Nm}/^\circ$) at $\Delta \varphi _{\text {lum,peak}}$	7.8 (− 2.6%)	8.0	8.2 (+2.6%)
Load sharing in $F_z$			
IVDs (%)	59.5	59.0	58.5
Ligaments (%)	24.4	22.2	19.7
Muscles (%)	16.1	18.8	21.8
Contribution to $M_y$			
IVDs (%)	34.5	34.8	35.1
Ligaments (%)	36.0	34.9	33.6
Muscles (%)	29.5	30.3	31.3

## Discussion

Humans differ in size, mass, strength, body composition and other mechanically related aspects. It is therefore relevant to understand, whether and to which extent the observed differences affect the internal mechanics and to build a mechanical model accordingly. Specifically for the human spine, subject-specific modelling could be one key to better understand the complex mechanics and dynamic interplay of the single parts. In recent years, subject-specific modelling has gained momentum and has reached an impressive level of individualisation already. Still, it remains a challenge. Alongside the inherent challenge of validation, which requests even better measurement techniques in the case of subject-specific models, we will discuss both currently published human spine models, the features and their level of validation and the potential of individualisation.

### Critical review of the developed models with respect to existing literature

The generic baseline model in this study is based on an already published human NMS spine model (Mörl et al. 2020), which was built using generic literature data for geometry and material characteristics and was validated using a custom-developed measurement device, revealing high confidence for the predicted total torque on L4/5 level. It was discussed that lever arms and, thus, attachment points of muscles and ligaments as well as zero lengths and slack lengths of ligaments are sensitive for a valid calculation of load sharing between passive muscle, ligaments and IVDs. In the Mörl et al. (2020) study, ligament’s stiffness had been reduced to the lowest possible literature values, but still found to be very stiff.

Our model is developed for forward-dynamic simulations, an approach rarely used in spine modelling. Even though forward-dynamics is considered to be more physiological (Pandy 2001), inverse-dynamic models are commonly used in spine research (Christophy et al. 2012; Cazzola et al. 2017; Overbergh et al. 2020)—some of them equipped with electromyography (EMG)-assisted methods to overcome limitations in solving the redundancy problem (Molinaro et al. 2020; Silvestros et al. 2022).

Inverse-dynamic simulations have the drawback of relying on accurate kinematic data. Marker-based optoelectronic approaches are considered the gold standard to characterise movement; however, applied on the spine, concerns persist over the accuracy of these skin-mounted techniques as high skin movement artefacts remain the major limitation in the correct assessment of vertebral motion (Zemp et al. 2014; Mahallati et al. 2016). Our forward-dynamic models, on the other hand, allow for a dynamic interplay of all modelled structures without the need of kinematic input, while 6 degrees of freedom (DOF) intervertebral joints account for translational and rotational movements at all spinal levels.

The main feature of our generic baseline model with respect to Mörl et al. (2020) is the full articulation between T1 and S1 and the separate modelling of IVDs, ligaments and muscles. In line with the full articulation, additional thoracic muscles were included and ligament properties revised. Further, we considered muscle deflections for long back muscles using an ‘via-ellipse’ algorithm (Hammer et al. 2019) instead of splitting the muscle into shorter muscle paths (Rupp et al. 2015) and considered the effect of intrinsic pressure in the nucleus pulposus.

Next to the generic baseline model, we developed an individualised model by adjusting the geometrical properties with respect to bone geometry and positions, muscle and ligament attachment points, and their respective length-dependent parameters to match the subject-specific dataset of Visible Human Male (VHM) (Spitzer et al. 1996). Both models were initiated with the same control mechanism to i) balance in an upright position (steady-state simulation) and ii) perform a forward flexion movement, stabilise in the flexed posture and return to their original state in upright position by using a forward-dynamic and purely muscle-driven approach.

**Fig. 9:**
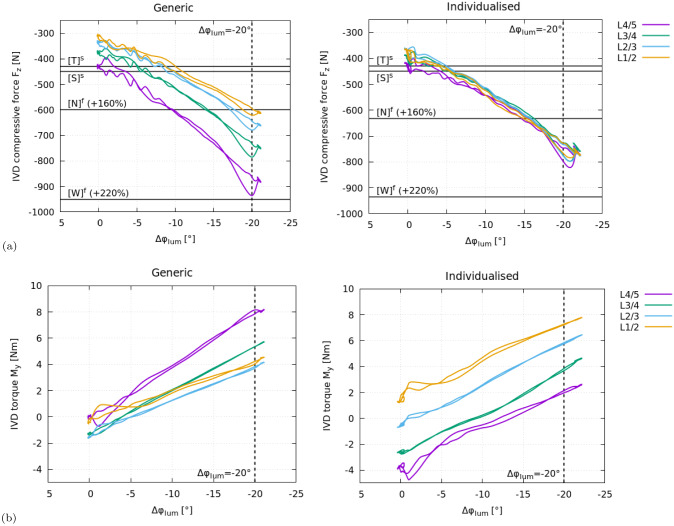
(**a**) Compressive load $F_z$ in local longitudinal direction and (**b**) torque in the sagittal plane $M_{y}$ for IVDs of the generic baseline (left) and subject-specific (right) model. Both compressing load and torque increase with spinal flexion. In both models, level L4/5 has borne the highest compressive load; however, the generic baseline model predicted a higher increase of compressive loading compared to the individualised model. Reported values for standing and literature-derived expected loading at flexion are indicated in (**a**) for both models (horizontal lines) with superscript ‘s’ and ‘f’, respectively. Key: [T]: Takahashi et al. (2006), [S]: Sato et al. (1999), [N]: Nachemson (1975), [W]: Wilke et al. (1999)

**Fig. 10:**
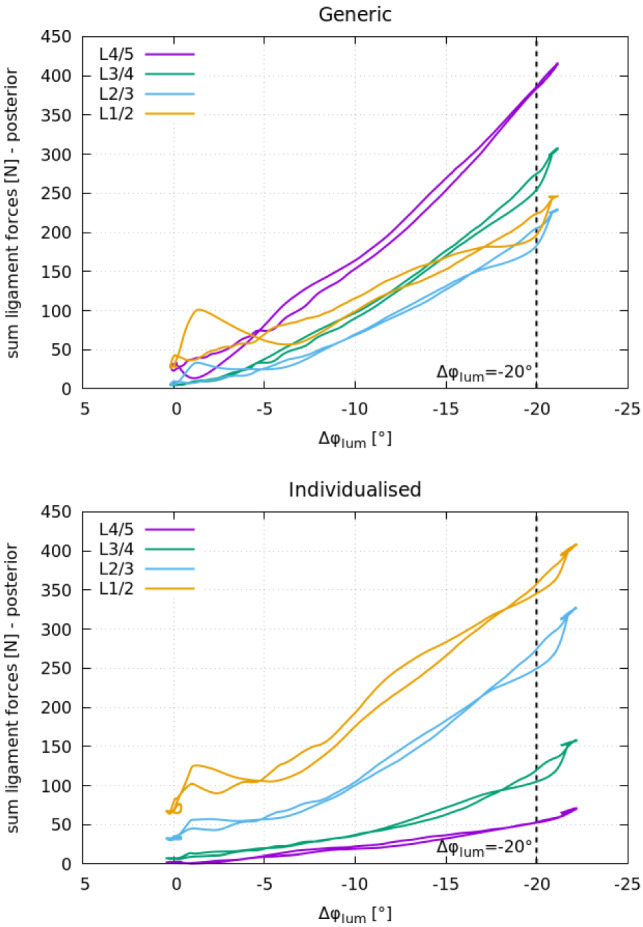
Cumulated forces of all posterior ligaments as a function of the change in lumbar angle $\Delta \varphi _{\text {lum}}$ over the course of the forward flexion movement for the generic baseline (top) and individualised model (bottom)

For the validation of computed IVD loads, experimental IDP measurements are commonly used to estimate compressive forces within the IVD. In standing posture, we compared our predicted compressive forces with the values of Nachemson and Elfström (1970), Sato et al. (1999) and Takahashi et al. (2006). Both the generic baseline and the individualised model in the equilibrated, steady-state position show very good agreement with values of $-374.3\,$N and $-394.7\,$N at IVD L3/4 and $-431.8\,$N and $-424.8\,$N at IVD L4/5, respectively (additionally, see Fig. 9a). Nachemson and Elfström (1970) report compressive forces between $-300.9\,$N and $-464.3\,$N. Similar values of $-430\,$N and $-450\,$N were derived from Takahashi et al. (2006) and Sato et al. (1999) (Subject No. 3) at L4/5, considering the disc area and a factor of 1.5 (Nachemson 1975) as values provided were measured in the nucleus pulposus. Note, Dreischarf et al. (2016) systematically reviewed experimental studies of the last decades measuring IDP at level L3/4 and L4/5 in standing position. However, we did not use the values reported in this review, because we found the unit conversion of the original values of Nachemson and Elfström (1970) in Dreischarf et al. (2016) to be incorrect. The respective values reported in Dreischarf et al. (2016) are between 20% and 40% too high.

For flexion movement, reported values for load increase on the lumbar IVDs vary. According to Nachemson (1975), forward flexion increases the load on the lumbar IVDs to 150–170%, according to Wilke et al. (1999) to 220%. Sato et al. (1999) reported a 270% average increase in load with flexion and Takahashi et al. (2006) reported 297% for a 20$^\circ$ flexion. In our simulations, compressive IVD loads increased to 196% and 188% on average in the lumbar spine with respect to the equilibrated position for the generic and individualised model, respectively. Thereby, our predicted results are between reported values by Nachemson (1975) and Wilke et al. (1999) as visualised in Fig. 9a.

Comparing the models’ local ($k_{\text {local,equ}}$, $k_{\text {local,peak}}$) and mean ($k_{\text {mean}}$) stiffnesses, the local stiffness in the equilibrated position $k_{\text {local,equ}}$ was very similar between the generic baseline and individualised model. During flexion and at flexion peak, the mean $k_{\text {mean}}$ and local stiffness $k_{\text {local,peak}}$, however, differed between the models. There were a $+60\%$ increase in $k_{\text {mean}}$ and a $+36\%$ increase in $k_{\text {local,peak}}$ with individualisation. We recall, for varying muscle co-contraction, we did not find a difference of such magnitude (Table 3). Moreover, with individualisation we found a shift in element contribution to the net joint force $F_z$ of $+73.4\%$ for muscles, $-83.3\%$ for ligaments and $+8\%$ for the IVD on L4/5 level (see Fig. 5) and a shift in element contribution to the net joint torque $M_y$ of $+183\%$ for muscles, $-84.8\%$ for ligaments, and $-74.4\%$ for the IVD (see Fig. 6), which is the largest difference found in all analysed levels (see Appendix Section 3 and 4 for other levels). At level L1/2, for example, the contributions remained almost the same.

Comparing the models’ contribution to the net joint torque $M_y$ (‘total’: red line, Fig. 6) to previously published experimental data  (Figure 5 Mörl et al. 2020), reveals that in both models the total torque at level L4/5 is in good agreement with Mörl et al. (2020), while in the generic baseline model all passive soft tissue elements together and the order of magnitude of their contributions to the functional spinal unit (FSU) stiffness are similar to Mörl et al. (2020), the individualised model’s predicted load distribution differed as discussed above.

With respect to the predicted trend in muscle recruitment in our simulations, we are in good agreement with experimentally acquired EMG data for unloaded flexion movements (Takahashi et al. 2006; Arjmand and Shirazi-Adl 2006). However, the validity of load distribution amongst ligaments and muscles in particular, remains unclear and requires further experimental studies.

A limitation of our models is the absence of intra-abdominal pressure (IAP). The increase of IAP during forward flexion and lifting tasks has shown to stabilise and unload the lumbar spine (Stokes et al. 2010; Hodges et al. 2005). In a recent study, Guo and colleagues demonstrated the IAP-induced spinal unloading effect during abdominal breathing in a flexed posture using a detailed generic spine model by considering a biomechanical representation of the abdominal cavity in combination with flexible multibody (MB) modelling of core muscles (Guo et al. 2021). The influence of IAP is not modelled in our model so far and should be considered in future work. Moreover, the arbitrary modelling of the facet joints, represented only in flexion movements by the incorporation of the CAP ligament, makes our models not suitable for extension movements. A modelling of the facet contact as in Damm et al. (2020) should be considered. Although, considering soft ligament properties and literature-derived prestrain values, our functional ligament stiffnesses, for the SSL ligament in particular, need further evaluation.

### The potential of subject-specific human spine models

The present study is the first study to directly compare two human whole spine models and quantify the potential differences between subject-specific geometries and generic values taken from the literature. As already discussed, we found different load sharing within the FSU. Different load sharing between two geometrically different models can be expected, but the quantification is important in our opinion, because both models could generally be used to predict internal load sharing for the same subject, here, the VHM during forward bending. The validated and published generic model might have been our choice for this task without subject-specific data available to individualise the model. And the choice might have seen appropriate, because VHM was reported to be nonpathological and with a height of 1.8 m and weight of 90 kg, which is close to the generic model values. Choosing the generic model, however, would have resulted in a large error in L4/5 level, see above.

**Fig. 12:**
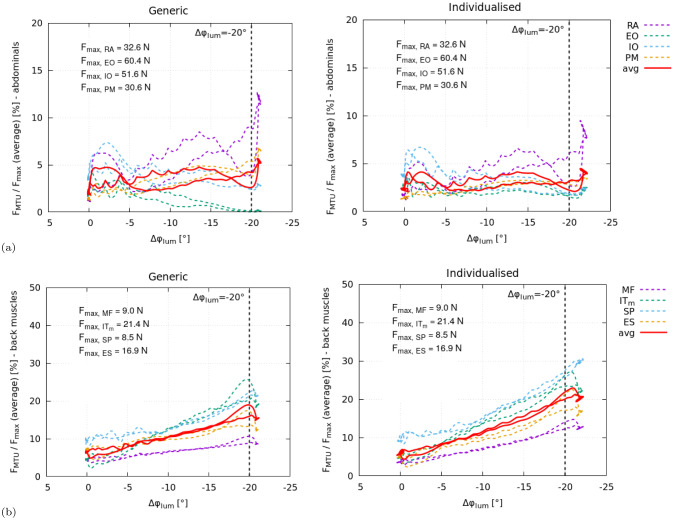
Computed **a** abdominal and **b** back muscle forces for a forward flexion movement as a function of the change in lumbar lordosis angle $\Delta \varphi _{\text {lum}}$ for the generic baseline (left) and individualised model (right). Forces in individual muscle threads were normalised with respect to their maximum isometric force $F_{\text {max}}$ and averaged over every major muscle group implemented in the model. Average over all muscle groups build the ‘avg’ (red line)

A subject-specific validation using subject-specific kinematics of bones, muscles and ligaments, material characteristics of muscles and ligaments, forces of muscles and ligaments and forces and torques of IVDs was not attempted in our contribution. However, the predicted differences between both model variants are plausible. First, the same model basis was used. Second, the same motor control for the settling procedure and the forward flexion was used and optimised for solving the muscle redundancy and low muscle stimulation (see Sect. 2.3). Third, the effective local joint stiffness $k_{local,equ}$ in the equilibrated position is similar between the models (see Sect. 3.1) in which active muscle contribution was low ($<6.5\%$ at $t_{\text {sim}}\approx 0\,s$, see Fig. 12). Muscle activity, however, increases with flexion, such that FSU stiffness towards higher flexion angles is higher. Thus, the observed differences are the result of dynamic interaction of all modelled elements and purely based on different geometries.

The present study strongly supports subject-specific modelling of human spines towards a better understanding of the mechanics. The path towards full subject-specific modelling includes geometry (Bruno et al. 2017; Overbergh et al. 2020); muscle characteristics (Bruno et al. 2017; Cazzola et al. 2017; Silvestros et al. 2022); ligament prestrains: currently open topic; IVD characteristics: currently open topic; individual movement pattern: currently open topic. Additionally, proper subject-specific validation is also needed, which is dependent on future measurement devices and techniques to validate the already many parameters of the model. Modelling the MTU, for instance, requires a large number of parameters. Extensive experimental studies are needed to determine these parameters on a subject-specific basis. Moreover, the tissue of interest usually needs to be dissected from its biological surrounding to investigate its characteristics; hence, parameters generally are originated from cadaveric measurements or animal studies. In contrast to the great amount of parameters required to model biomechanical systems, only limited data for such parameters are available in literature leaving alone the possibility to personalised parameters. In the present study, we have used CT-derived geometric information to build an individualised model of the VHM. Muscle-specific lengths ($l_{\text {CE,opt}}$, $l_{\text {SEE,0}}$) and ligament parameters ($F_A$, $F_B$, $\epsilon _A$, $\epsilon _B$) were scaled to the geometry ($l_{\text {MTU}}$, $l_{\text {LIG,0}}$). For the latter, personalised stiffness properties were reached by considering individual ligament lengths. Muscle parameters such as the PCSA of muscles, were not adjusted to the subject.

In our opinion, generic models will still play an important role in understanding general mechanics of the human spine. However, improved subject-specific approaches will be crucial to improve the knowledge on inter-individual characteristics of sub-populations, e.g. adolescents, elderly, male, female, etc., or to study pathologies, e.g. spine deformity, muscle asymmetry, etc.

### Supplementary Information

Below is the link to the electronic supplementary material.

Supplementary file 1 (pdf 793 KB)

## Figures and Tables

**Fig. 7:**
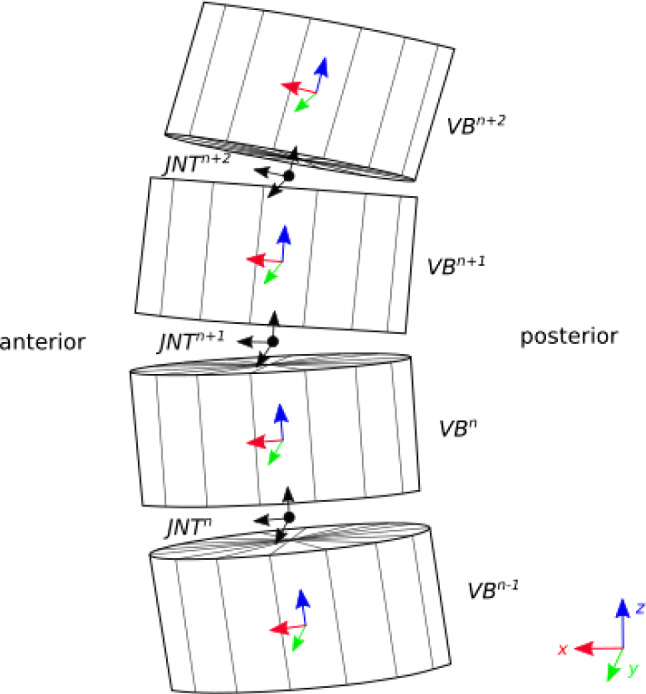
Illustration of the joint-body configuration and axis convention in our multibody (MB) spine models. Next to local, body-fixed coordinate systems $\text {VB}^n$, positioned in the COM of vertebrae *n*, joint coordinate systems $\text {JNT}^n$ are defined, whose location and orientation are fixed to their respective subjacent vertebra $\text {VB}^{n-1}$

**Fig. 8:**
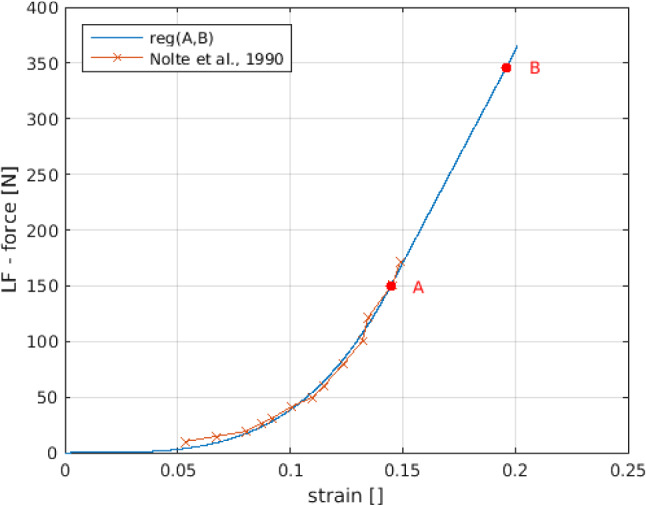
Force–strain characteristics for the LF ligament with the optimisation-derived value pairs A$(\varepsilon _A,F_A)$ and B$(\varepsilon _B,F_B)$ indicated

**Fig. 11:**
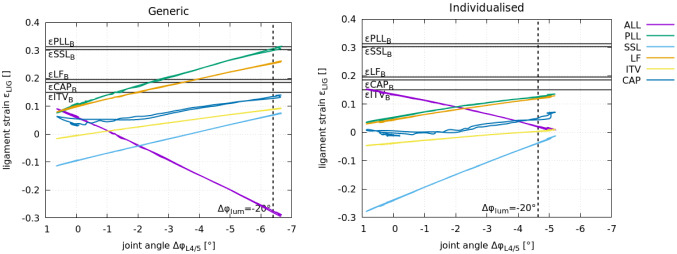
Strain of individual ligaments at level L4/5 as a function of the change in joint angle $\Delta \varphi _{L4/5}$ in the generic baseline (left) and individualised model (right). The ligaments’ critical threshold before failure $\varepsilon _B$ is indicated. Starting in a equilibrated, prestrained state, ligament strain increases with spinal flexion for all posterior ligaments, while the anterior ligament ALL becomes slack. The unstrained ITV and SSL is due to reported or missing literature data (Sect. 2.1.4)

**Fig. 13:**
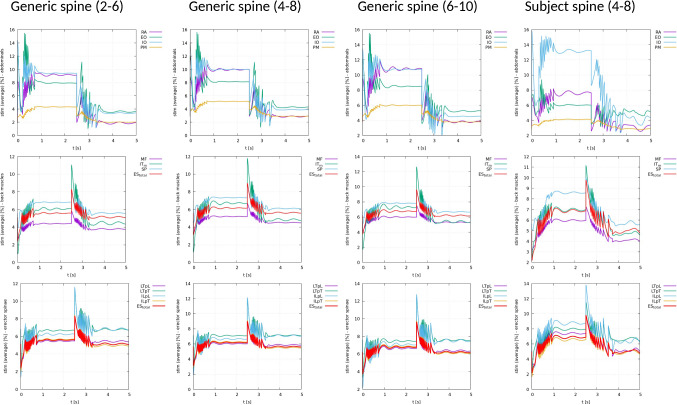
Muscle stimulations predicted to drive forward flexion-to-extension in the generic baseline model used (4–8) in this study including two variants of co-contraction (2–6, lower co-contraction and 6–10, higher co-contraction) and the subject-specific, individualised spine model (4–8) used in this study. The stimulation values never exceed 16% of maximum stimulation. 2–6 means $u_{open}=2\%/6\%$ or 0.02/0.06, respectively. The total contribution of $u_{open}$ to *u* is 50%, see Sect. 2.3

## Data Availability

The presented mathematical algorithms were implemented in our in-house multibody (MB) simulation framework **demoa** (Schmitt 2022), if not otherwise stated. **demoa** is a C/C++-based framework under constant development since almost 20 years now. It includes a preprocessor **calcman** for the calculation and scaling of three-dimensional (3D) anthropometric data (Rupp et al. 2015); an equation generator and simulator **sim** and an animation tool **ani**. **demoa** and the generic spine model (Hammer et al. 2022) presented herein are available open-source http://get-demoa.com. The subject-specific model is available upon request.

## Appendix 1: Definition of coordinate systems and evaluation criteria

The global reference frame in our spine models is located in the COM of the pelvis. The global $x-$axis of the Cartesian coordinate system points in ventral direction, the $y-$axis points left and the $z-$axis is longitudinal to the spinal column pointing in cranial direction (Fig. 7). This axis convention is maintained for other local, i.e. body and joint coordinate systems.

Figure 7 illustrates the joint-body configuration of our spine models. The body-fixed coordinate systems $\text {VB}^n$ are located in the COM of every body *n* starting with the pelvis as the first body of the kinematic chain. From there, an alternating joint-body configuration is pursued up to the first thoracic vertebra (T1) with intervertebral joints connecting adjacent VBs. Thereby, every joint coordinate system ${\text{JNT}}^{{\text{n}}}$ is defined from a proximal and distal end using a relative displacement and rotation with respect to its subjacent vertebra $\text {VB}^{n-1}$ and superjacent vertebra $\text {VB}^n$. Note, deriving the location and rotation of the global VB and joint coordinate systems differs between the models. The reader is referred to Appendix 2 and Appendix 3 for a detailed description of the methods applied in the generic baseline and individualised model, respectively.

For the definition of our models’ equilibrated position we considered sagittal balance as a criterion for stable upright stance. However, since our models do not incorporate the head, cervical spine and arms, the commonly used plumb line from the posterior–superior edge of S1 to the last cervical vertebra (C7), is not applicable. Instead, we focussed on the gravitational line of thorax masses to lie slightly posterior to the femoral head according to Roussouly et al. (2006) and Schwab et al. (2006). In our spine models, hence, the plumb line for sagittal balance passes through the COM of T5 ($\pm 0.5$ mm) in the initial and equilibrated position, which is ensured by slightly adjusting the pelvic angle in the sagittal plane.

The lumbar lordosis angle $\varphi _{\text {lum}}$ and the thoracic kyphosis angle $\varphi _{\text {tho}}$ are typically representative for the evaluation of spinal curvature. The lumbar angle $\varphi _{\text {lum}}$ was measured between the vertebra L1 and L5 using the angular difference in vertical $z-$axis orientation of the VBs according to Polly et al. (1996) when projected onto the sagittal plane ($x-z-$plane), while $\varphi _{\text {tho}}$ was measured between T1 and L1. We will take use of these characteristic curvatures in the display of results.

## Appendix 2: Generic spinal geometry

Individual VB locations and orientations were derived from the Kitazaki description for a generic spinal curvature of a ‘normal posture’ (Table 1 Kitazaki and Griffin 1997) during upright stance. A polynomial fit to the reported spinal curvature was used to determine the *n*-th VB position in ventral direction $VB^n_x$ as a function of their respective axial position $VB^n_z$ as well as their orientation in the sagittal plane $\theta ^n$ such that the local longitudinal axis of each VB frame is tangent to the function describing spinal curvature.

6 $$\begin{aligned} VB^n_x= & {} 74.33\cdot (VB^n_z)^6 -153.55\cdot (VB^n_z)^5\nonumber \\{} & {} +113.49\cdot (VB^n_z)^4-34.25\cdot (VB^n_z)^3\nonumber \\{} & {} +3.12\cdot (VB^n_z)^2 +0.24\cdot VB^n_z\nonumber \\{} & {} +0.0003 \end{aligned}$$

7 $$\begin{aligned} \theta ^n= & {} 6\cdot 74.33\cdot (VB^n_z)^5 -5\cdot 153.55\cdot (VB^n_z)^4\nonumber \\{} & {} +4\cdot 113.49\cdot (VB^n_z)^3-3\cdot 34.25\cdot (VB^n_z)^2\nonumber \\{} & {} +2\cdot 3.12\cdot VB^n_z +0.24 \end{aligned}$$

Note that the a sixth-order polynomial was chosen to fit data of the entire spinal column, though the cervical spine is omitted in this work. $VB^n_z$ were directly taken from Kitazaki and Griffin (1997). Eventually, the whole spinal column was rotated about the lateromedial axis through the sacrum S1 by an angle of 10 degrees to compensate for the apparent forward tilt in the original Kitazaki spine and to fulfil our criterion of sagittal balance (see Appendix 1 for more details). The VB locations of the Kitazaki spine were then linearly scaled along the caudocranial and dorsoventral axis to match the desired total trunk length for a 50th percentile male as mentioned in Sect. 2.1.1.

Note that for the calculation of inertial effects VBs were assumed to be ideal cylinders with a homogeneous bone density of $1.5 ~\mathrm {g/cm^{3}}$. VB dimensions (height, width and depth) are based on multiple datasets from the literature and were inherited from our previous model (Mörl et al. 2020; Rupp et al. 2015). All body locations and orientations are listed in Table 4.

Joints were defined in the geometric centre of the area enclosed by adjacent vertebral endplates completing the kinematic chain of the detailed thoracolumbar spine; their orientation resulted from the average orientation of both adjacent VB frames (Rupp et al. 2015). Throughout this work, we refer to this literature-derived geometry as the model’s initial position that we used as a reference to derive the muscles’ and ligaments’ characteristic lengths.

**Table 4:** VB locations and orientations in the initial position of the generic baseline model

Level	$x\,\left[ \textrm{cm}\right]$	$z\,\left[ \textrm{cm}\right]$	$\theta \,\left[ ^\circ \right]$
T1	−1.31	46.92	9.0
T2	−1.67	44.77	10.1
T3	−2.05	42.65	10.1
T4	−2.42	40.49	8.9
T5	−2.71	38.34	6.6
T6	−2.91	36.10	3.2
T7	−2.95	34.07	−0.7
T8	−2.85	31.93	−5.1
T9	−2.56	29.74	−9.5
T10	−2.10	27.46	−13.2
T11	−1.50	25.11	−15.4
T12	−0.85	22.81	−15.6
L1	−0.18	20.26	−13.3
L2	0.36	17.45	−8.0
L3	0.59	14.39	−0.4
L4	0.38	11.12	6.9
L5	−0.11	7.86	9.2
S1	−0.44	5.17	3.4
Pelvis	0	0	0

Positions *x* and *z* from Kitazaki and Griffin (1997) were scaled to the dimensions of a 50th percentile male according to Laubach et al. (1978). Vertebra orientations in the sagittal plane, denoted by angle $\theta$, were set tangent to the spinal curve polynomial

## Appendix 3: Generating individualised model geometries

For the current study, we generated a subject-specific (MB) spine model based on anatomical data from 3D (CT) scans. Together with the custom-developed image processing software, Dicomtilt (Little and Adam 2015), we partly automatised the assembly of the kinematic chain based on information about vertebral endplate positions and orientations.

In particular, we determined the COM of the VB $\vec {P}_{\text {VB}}^i$ for every vertebra *i* (with $i=$ S1,...,T1) in global coordinates as the arithmetic mean of the global positions of the superior $\vec {P}^{\text {sup},i}_{\text {EPC}}$ and inferior endplate centroids $\vec {P}^{\text {inf},i}_{\text {EPC}}$. At the same time, these points served as origins of local VB coordinate systems. Intervertebral joint coordinate systems $\vec {P}^{i}_{\text {JNT}}$ were defined by the arithmetic mean of the inferior endplate centroid of the superjacent vertebra $\vec {P}^{\text {inf},(i+1)}_{\text {EPC}}$ and the superior endplate centroid of the subjacent vertebra $\vec {P}^{\text {sup},i}_{\text {EPC}}$.

8 $$\begin{aligned} \vec {P}_{\text {VB}}^i= & {} \frac{\vec {P}^{\text {sup},i}_{\text {EPC}}+\vec {P}^{\text {inf},i}_{\text {EPC}} }{2} \end{aligned}$$

9 $$\begin{aligned} \vec {P}^{i}_{\text {JNT}}= & {} \frac{\vec {P}^{\text {sup},i}_{\text {EPC}}+\vec {P}^{\text {inf},(i+1)}_{\text {EPC}} }{2} \end{aligned}$$

Note the index *i* ranges from L5/S1 to T1/2 when enumerating joints.

Similarly, the orientation of the local VB coordinate systems was considered to be the average orientation of two corresponding vertebral endplates with respect to the global reference frame. We defined the average orientation of the two coordinate systems of the lower $\mathcal {O}^{\text {inf},i}$ and the upper endplate $\mathcal {O}^{\text {sup},i}$ via axis-angle representations of their rotation matrices $R^{\text {inf},i}$ and $R^{\text {sup},i}$ as described in Hammer et al. (2021). First, we calculated the relative rotation matrix $R^{{\text {inf}}\rightarrow {\text {sup}},i}$ from $\mathcal {O}^{\text {inf},i}$ and $\mathcal {O}^{\text {sup},i}$ and its associated rotation axis $\vec {a}^{i}$ and rotation magnitude $\theta _a^i$ about this axis (with $\theta _a^{i}\in \left[ 0,\pi /2\right]$ in this context) as follows:

10 $$\begin{aligned} R^{{\text {inf}}\rightarrow {\text {sup}},i}&= (R^{\text {inf},i})^{-1}\cdot R^{\text {sup},i} \end{aligned}$$

11 $$\begin{aligned} \theta _a^i&= \arccos \left( \frac{\textrm{Tr} (R^{{\text {inf}}\rightarrow {\text {sup}},i})-1}{2}\right) \nonumber \\ \vec {a}^i&= \frac{1}{2\cdot \sin \theta } \end{aligned}$$

12 $$\begin{aligned}&\cdot \left( \begin{array}{c} R^{{\text {inf}}\rightarrow {\text {sup}},i}(3,2)-R^{{\text {inf}}\rightarrow {\text {sup}},i}(2,3)\\ R^{{\text {inf}}\rightarrow {\text {sup}},i}(1,3)-R^{{\text {inf}}\rightarrow {\text {sup}},i}(3,1) \\ R^{{\text {inf}}\rightarrow {\text {sup}},i}(2,1)-R^{{\text {inf}}\rightarrow {\text {sup}},i}(1,2)\end{array}\right) ~, \end{aligned}$$

where $R^{{\text {inf}}\rightarrow {\text {sup}},i}(j,k)$ denotes the matrix entry in row *j* and column *k* ($j,k=\{1;2;3\}$) and Tr($R^{{\text {inf}}\rightarrow {\text {sup}},i}(j,k)$) the matrix’ trace. Second, the orientation of the *i*-th VB coordinate system $\mathcal {O}_{\text {VB}}^i$ relative to $\mathcal {O}^{\text {inf},i}$ was found by rotating about the same axis $\vec {a}^i$ as above by only half the magnitude compared to the rotation necessary to reach $\mathcal {O}^{\text {sup},i}$, i.e. by $\theta _a^i/2$. Then, the rotation matrix $R^{{\text {inf}}\rightarrow {\text {VB}},i}$ was calculated following the Rodrigues’ rotation formula

13 $$\begin{aligned} R^{{\text {inf}}\rightarrow {\text {VB}},i}&= I + \sin \left( \frac{\theta _a^i}{2}\right) \cdot A^{i} \nonumber \\&\quad + \left( 1-\cos \left( \frac{\theta _a^i}{2}\right) \right) \cdot \left( A^i\right) ^2 \end{aligned}$$

with $A^i$ denoting the skew-symmetric matrix of the rotation axis $\vec {a}^i$,

14 $$\begin{aligned} A^i= & {} \left( \begin{array}{c c c} 0 &{} -\vec {a}^i(3) &{} \vec {a}^i(2)\\ \vec {a}^i(3)&{} 0 &{} -\vec {a}^i(1)\\ -\vec {a}^i(2) &{} \vec {a}^i(1) &{} 0 \end{array}\right) \quad . \end{aligned}$$

Afterwards, this rotation matrix given in local coordinates of the corresponding lower endplate system $\mathcal {O}^{\text {inf},i}$ was transformed back into the global reference frame,

15 $$\begin{aligned} R_{\text {VB}}^i=R^{\text {inf},i}\cdot R^{{\text {inf}}\rightarrow {\text {VB}},i} \quad , \end{aligned}$$

resulting in the VB orientations $R_{\text {VB}}^i$ in the global reference frame. Analogue to that, the orientation of joint coordinate systems $R_{\text {JNT}}^i$ was determined by the average rotation matrix of the upper endplate of the inferior vertebra $\mathcal {O}^{\text {sup},i}$ and the lower endplate of the superior vertebra $\mathcal {O}^{\text {inf},(i+1)}$ enclosing the intervertebral space. After the global VB and joint positions and orientations were determined, the kinematic chain for the MB model was assembled by transformation into local coordinates.

Every intervertebral joint *i* was defined from a proximal and a distal end using four-dimensional joint triads containing the relative displacement vector $\vec {p}^{~i}_{\text {JNT}}$ and rotation matrix $r^{i}_{\text {JNT}}$ with respect to adjacent VBs. The proximal ($\vec {p}^{\ \text {prox},i}_{\text {JNT}}, r^{\text {prox},i}_{\text {JNT}}$) and distal ($\vec {p}^{\ \text {dist},i}_{\text {JNT}}, r^{\text {dist},i}_{\text {JNT}}$) local representation of the intervertebral joint, hence, can be computed as follows:

16 $$\begin{aligned} \vec {p}^{\ \text {prox},i}_{\text {JNT}}= & {} (R_{\text {VB}}^i)^{-1}\cdot (\vec {P}^i_{\text {JNT}}-\vec {P}^i_{\text {VB}}) \end{aligned}$$

17 $$\begin{aligned} r^{\text {prox},i}_{\text {JNT}}= & {} (R_{\text {VB}}^{i})^{-1}\cdot R^i_{\text {JNT}} \end{aligned}$$

18 $$\begin{aligned} \vec {p}^{\ \text {dist},i}_{\text {JNT}}= & {} (R_{\text {VB}}^{i+1})^{-1}\cdot (\vec {P}^i_{\text {JNT}}-\vec {P}^{i+1}_{\text {VB}}) \end{aligned}$$

19 $$\begin{aligned} r^{\text {dist},i}_{\text {JNT}}= & {} (R_{\text {VB}}^{i+1})^{-1}\cdot R^{i+1}_{\text {JNT}} \end{aligned}$$

with the global position and orientation of the superjacent ($\vec {P}^{i+1}_{\text {VB}}$, $R_{\text {VB}}^{i+1}$) and subjacent ($\vec {P}^{i}_{\text {VB}}$, $R_{\text {VB}}^{i}$) vertebra, respectively. By overlapping the orthonormal axes of proximal and distal joint triads starting from S1 to T1, the joint-body configuration of the subject-specific multibody (MB) model is fully defined.

Finally, the vertebra widths were estimated from selected landmarks on the superior and inferior endplates, while the distance between corresponding endplate centroids defined the vertebra heights in order to approximate the volume and to compute mass and inertia of the individual vertebral cylinders by assuming a homogeneous bone density of $1.5~\mathrm {g/cm^3}$. Note the VB mass was multiplied by a factor of 1.5 to account for the weight of spinal processes and the vertebral arch.

To this end, we take the additional note that special attention has to be drawn on appropriate angle transformation when using software with different angle representations. In the anatomical landmark output from the CT data, the incline of the endplate plane was represented by angles in the sagittal $\theta _{sag}$ and coronal plane $\theta _{cor}$; the axial rotation was described by the facet rotation $\theta _{slew}$ about the normal vector to the inclined endplate plane. In contrast to these angles arising from projections onto specific planes, rotations in our multibody (MB) framework are expressed in Cardan angles, while axis angles were adopted in the IVD force–displacement function and measurement, and to calculate average orientations. Hence, we converted all different sets of angles into the more universal rotation matrices.

**Table 5:** Thoracolumbar ligament parameters. Pairs of force–strain values at the characteristic points A$(\varepsilon _A,F_A)$ and B$(\varepsilon _B,F_B)$ were determined for every ligament by taking the thoracolumbar average (av.) value or through optimisation (opt.) from Nolte et al. (1990). Determined forces $F_{A}$ and $F_{B}$ were divided by three to comply with Mörl et al. (2020) and divided by the number of threads *l*. Here, the effective values for every ligament are shown (sum of all threads *l* representing a ligament)

Ligament	$F_A/3~\left[ \textrm{N}\right]$	$\varepsilon _A~\left[ ~\right]$	$F_B/3~\left[ \textrm{N}\right]$	$\varepsilon _B~\left[ ~\right]$	*l*	Reference
ALL (av.)	16.25	0.1188	138.54	0.4563	3	Chazal et al. (1985)
PLL (av.)	15.0	0.105	111.88	0.3138	1	Chazal et al. (1985)
SSL (av.)	11.85	0.1227	59.85	0.3036	1	Chazal et al. (1985)
ITV (av.)	6.67	0.085	29.17	0.15	1	Chazal et al. (1985)
LF (opt.)	49.95	0.1446	115.35	0.1960	3	Nolte et al. (1990)
CAP (opt.)	43.33	0.1430	100.61	0.1858	1	Nolte et al. (1990)

## Appendix 4: Ligament parameters

Our nonlinear ligament force function $f_{\text {LIG}}$ adapted from Günther et al. (2007) is defined by a pair of characteristic points A$(l_A,F_A)$ (nonlinear to linear transition) and B$(l_B,F_B)$ (state before failure).

For the ALL, PLL, SSL and ITV ligament, these values were derived using force–strain data from Chazal et al. (1985) (see Table 5). For the LF and CAP ligament, the force–strain value pairs A$(\varepsilon _A,F_A)$ and B$(\varepsilon _B,F_B)$ were determined by an optimisation algorithm (MATLAB R2018b, The Mathworks, Natick, MA, USA) in which the optimal regression curve $\text {reg}(A,B)$ was found to initial literature data from Nolte et al. (1990). The parameters of the defined objective function were found using the integrated default interior-point algorithm of *fmincon*, i.e. by minimising the squared error $\parallel f_{\text {LIG}}\left( A,B\right) \,-\text {reg}_{n}\left( A,B\right) \parallel _{2}\;\rightarrow \,\text {Minimum}$ between the polynomial $\text {reg}_n(A,B)$ and the function $f_{\text {LIG}}(A,B)$. Starting values for A$(\varepsilon _A,F_A)$ and B$(\varepsilon _B,F_B)$ as well as the lower and upper bounds of $F_A$ and $F_B$ were estimated from initial data points. Lower and upper bounds for $\varepsilon _A$ and $\varepsilon _B$ were set to moon parameters.

The resulting force–strain regression curve is depicted for the LF ligament, in Fig. 8 as a function of optimisation-derived parameter pairs A$(\varepsilon _A,F_A)$ and B$(\varepsilon _B,F_B)$ listed in Table 5. The required characteristic lengths $l_A$ and $l_B$ in our ligament model were derived from obtained strain values multiplied with the individual thread length of each ligament.

## Appendix 5: IVD forces and torques

In Fig. 9, compressive load (negative force in local $z-$axis) in (9a) and the torque in the sagittal plane (around $y-$axis) in (9b) are depicted for the lumbar IVDs as a function of the change in lumbar lordosis angle $\Delta \varphi _{\text {lum}}$ predicted by both models. As observed earlier in Sect. 3.1, in the equilibrated position that is reflected in Fig. 9a at $\Delta$ $\varphi _{\text {lum}}\approx 0\,^\circ$, compressive load was 9.2% higher in sum in the individualised model than in the generic baseline model, which can be attributed to the 17% higher trunk weight in the individualised model.

With forward bending, compressive load increased caudally with the generic baseline model experiencing a slightly higher average increase of 96% opposed to the 88% average increase in the individualised model resulting in comparable absolute loading conditions between the models. Moreover, in both models IVD L4/5 bore the highest compressive load at $\Delta \varphi _{\text {lum}}=-20^\circ$ with $F_{z,L4/5}^{\text {generic}}=-864.4~\textrm{N}$ and $F_{z,L4/5}^{\text {subject}}=-746.4~\textrm{N}$ in the generic baseline and individualised model, respectively. Note the full articulation in combination with the discrete modelling of single IVDs (bushing elements) has advanced the model’s capability of simulating realistic thoracolumbar kinetics and kinematics. In previous publications (Mörl et al. 2020; Rupp et al. 2015) the nonlinear lumbar IVD model presented in Karajan et al. (2013) was used; however, it comprised the generalised response of a single lumbar IVD (L4/5). Moreover, by considering intrinsic IVD pressure in the nucleus pulposus, we modified the initial state of the IVD compared to our previous models’ zero-displacement approach by applying an additional offset force in $z-$direction. This has to be considered in the interpretation and comparison of results.

Regarding the IVD torque in the sagittal plane (Fig. 9b), at the beginning of the simulation ($\Delta$ $\varphi _{\text {lum}}\approx 0\,^\circ$) rotational loading was higher in the individualised model than in the generic baseline model (Sect. 3.1). With increasing spinal flexion, anterior torque acting on the lumbar IVDs increased and at some levels; hence, loading transited from posterior (negative) to anterior (positive) torque given the increasing global forward tilt of IVDs with spinal flexion. At $\Delta \varphi _{\text {lum}}=-20^\circ$, rotational loading was comparable between the models with IVD L4/5 bearing the highest torque of $M_{y,L4/5}^{\text {generic}} = 7.9~\textrm{Nm}$ in the generic baseline model compared to $M_{y,L1/2}^{\text {subject}} = 7.2~\textrm{Nm}$ at IVD L1/2 in the individualised model. After all, the relative change in IVD torque was higher in the generic baseline model where spinal flexion increased rotational loading in sum by a factor of 6.5 compared to the individualised model that recorded 2.3-times higher torques in sum at $\Delta \varphi _{\text {lum}}=-20^\circ$.

## Appendix 6: Ligament forces and strain

In a forward bending movement, posterior ligaments are stretched, while anterior ligaments become slack. Accordingly, forces in the anterior ligaments, i.e. ALL, became negligibly small in our simulations ($<0.3~\textrm{N}$) and, hence, were neglected in the display of results. The cumulated forces of all posterior ligaments, i.e. PLL, SSL, LF, CAP and ITV, in Fig. 10 revealed an increasing restoring force with increasing flexion. At $\Delta \varphi _{\text {lum}}=-20^\circ$, the highest stress was borne at level L4/5 with $F_{\text {LIG},L4/5}^{\text {generic}}=386.8~\textrm{N}$ in the generic baseline and at level L1/2 in the individualised model, respectively, with $F_{\text {LIG},L1/2}^{\text {subject}}=358.3~\textrm{N}$.

Generally speaking, posterior ligament forces were on average 1.3-times higher in the generic baseline model. Multiple factors were determined as most vital in contributing to this discrepancy. Firstly, the orientation of the CAP ligament largely influenced its contribution to longitudinal strain. In our generic baseline model the resulting CAP line of action appeared to be more vertical than in the individualised model, thus bearing more longitudinal stress during forward flexion. Secondly, albeit identical ligament input parameters in both models (Table 5), the ligaments’ stiffness depends on the initial ligament rest length that in turn depends on the respective ligament modelling and on the model’s geometry (initial position), i.e. spinal curvatures, vertebral anatomy and IVD height.

That being said, ligaments in the individualised model had on average a 1.4-times longer rest length in their respective initial position than ligaments in the generic baseline model. Firstly, higher intervertebral spaces (IVD height) between corresponding vertebral endplates were observed for the segmented geometry of the Visible Human Male (VHM), thus resulting in longer ligament rest lengths in our individualised model. Secondly, in case of the LF ligament, different modelling in the applied finite element methods to derive the individualised model was responsible for significantly longer rest lengths than in the generic baseline model as the superior edge of the vertebral arch served as both insertion and origin point. In our strain-based approach, this results in overall softer ligament properties in the individualised model. The third factor worth mentioning is that the dynamic interaction of the models’ initial geometry (VB orientation) and model-specific muscle and ligament lever arms together impact the gravitational settling and, hence, the manifestation of spinal curvatures in the equilibrated position which, in turn, influences the ligaments’ length and strain.

In Fig. 11, the strain $\varepsilon _{\text {LIG}}$ of individual ligaments is depicted as a function of the change in the L4/5 joint angle $\Delta \varphi _{L4/5}$ for the generic baseline (left) and individualised model (right). Note strain–angle relations predicted for all individual lumbar joint levels can be found in Section 5 of the Supplementary data. Confirmed by both models, the strain of all posterior ligaments increased with spinal flexion, while the anterior ligament ALL became slack. The strain was computed according to $\varepsilon _{\text {LIG}}= (l_{\text {LIG}}-l_{\text {LIG},ref})/l_{\text {LIG},ref}$ with the reference rest length $l_{\text {LIG},ref}=l_{\text {LIG},0}/(1+\varepsilon _{\text {LIG,init}})$. The ligaments’ initial rest length $l_{\text {LIG},0}$ was taken from the models’ respective literature-derived and CT-derived geometry and $\varepsilon _{init}$ was derived from the literature data as elucidated in Sect. 2.1.4.

In the generic baseline model, all ligaments started with an equilibrated prestrain of 3–11% into the flexion movement at $\Delta \varphi _{L4/5}\approx 0^\circ$, apart from the ITV and SSL that already had a zero or negative initial prestrain $\varepsilon _{\text {LIG,init}}$ prescribed in their initial position. With spinal flexion, all ligaments were subjected to moderate strains within the linear portion of the ligaments’ stress–strain curve ($<\varepsilon _B$) except for the LF ligament that was predicted to be overloaded at $\Delta \varphi _{L4/5}\approx -4^\circ$ which corresponds to a lumbar flexion angle of $\Delta \varphi _{lum}\approx -12^\circ$.

In the individualised model, strains were generally smaller compared to the generic baseline model apart from the ALL which experienced an increase in prestrain during the settling process. This may be partly attributed to the free model-specific settling dynamics and can be considered to be subject-specific. In case of the SSL, however, the individualised model has shown a significantly high negative equilibrated prestrain at L4/5. According to Adams et al. (1980), the SSL transition from slack to strained is at $3^\circ$ lumbar joint angle. Regarding the strain–angle relation, this is well represented in the generic baseline model. In the individualised model, however, the SSL does not reach the strained state for the whole flexion movement, despite a maximum joint angle of $\Delta \varphi _{L4/5}<-5^\circ$. Thus, regarding the individualised model, the initial prestrain chosen for the SSL is certainly too small at level L4/5 but shows feasible strain–angle relations in other lumbar levels (see Section 5 Supplementary data). Considering an initial prestrain of -6% or altering the strongly age-dependant mechanical properties (Nachemson and Evans 1968; Tkaczuk 1968) would be advisable to better represent the SSL in this subject. However, optimising ligament parameters for all lumbar levels requires a highly repetitive process that is beyond the scope of this study. With spinal flexion, ligament strains increased to moderate values in the individualised model but remained in a physiologic range, i.e. did not increase beyond the critical threshold $\varepsilon _B$.

Overall, the predicted contribution of passive structures (IVDs + ligaments) at level L4/5 to $F_z$ and $M_y$ well corresponds to (Figure 5 and 6 Mörl et al. 2020) despite using a linear IVD bushing element in this study and considering an active flexion movement with the contribution of active muscle forces.

## Appendix 7: Muscle forces

In Fig. 12, forces of the MTU ($F_{\text {MTU}}$) to the simulated flexion-to-extension movement (Figure 4) normalised with respect to their respective maximum isometric force $F_{\text {max}}$ (see Supplementary data, Tables 2 and 3) and averaged over all threads of a muscle group, are visualised for selected abdominal muscles (RA, EO, IO, PM) in (Fig. 4a) and back muscles (MF, ES, IT, SP) in (Fig. 4b) as a function of the change in lumbar angle.

In the equilibrated position at $\Delta \varphi _{L4/5}\approx 0^\circ$ it required a persistent activation of the back muscles in particular with 6.5% and 5.8% of their respective $F_{\text {max}}$ in the generic baseline and individualised model, respectively, to stabilise the fully articulated spine in an upright posture. Abdominals contributed with approximately 2% of their $F_{\text {max}}$ in both models in the equilibrated position.

An increase in abdominal activation associated with a short decrease in back muscle support initiated the forward flexion of the spine. Results of the simulation have shown that with forward flexion the support of back muscles increases, while the force generated in the abdominal muscles stays nearly constant. The controller-caused fluctuations in muscle forces can be observed every time the model comes close to reaching the desired target position and the control algorithm switches to the next target position (Sect. 2.3).

At flexion peak, back muscles contributed with an average 16.0% and 20.1%, while abdominals contributed with 4.2% and 3.2% of their respective $F_{\text {max}}$ in the generic baseline and individualised model, respectively. The deviance in back muscle support may be attributed to the higher trunk weight in the individualised model. Overall, the long SP muscles contributed most during forward flexion in both models, while the short IT muscles were found to be of significant importance to spinal stability. For the majority of flexion movement, abdominals contributed on average under 5% of their $F_{\text {max}}$ in both models.

## Appendix 8: Muscle stimulations

Figure 13 shows the corresponding muscle stimulations for the reported load case: forward flexion extension. Depicted is the generic model (4–8, used in the manuscript) including two variants of co-contraction (2–6, lower and 6–10. higher) and the subject-specific spine model (4-8). Note, depicted co-contraction values, for example, 2-6 represent $u_{open}=2\%/6\%$, which are effectively $1\%/3\%$ or 0.01/0.03 of *u* (see Sect. 2.3 for further details). The stimulation values never exceed 16% of maximum stimulation. We prefer showing muscle stimulation over muscle activity, because stimulation is similar to EMG. Note: We solve a dynamic system and use muscle stimulation to drive the model and do not distribute calculated kinematics and dynamics among the present elements to solve static optimisation.
