# Supplementary material for: Effects of geometric individualisation of a human spine model on load sharing: neuro-musculoskeletal simulation reveals significant differences in ligament and muscle contribution
Source: Biomech Model Mechanobiol. 2023 Jan 5;22(2):669–94. doi: 10.1007/s10237-022-01673-3 (PMC10097810; doi:10.1007/s10237-022-01673-3)
Supplement: Supplementary file 1 — (pdf 793 KB) [file 10237_2022_1673_MOESM1_ESM.pdf]

Supplementary Data

Effects of geometric individualisation of a human spine model on load sharing: neuro-musculoskeletal simulation reveals significant differences in ligament and muscle contribution

Biomechanics and Modeling in Mechanobiology

Laura Meszaros-Beller[1,2], Maria Hammer[2,3], Julia M Riede[2], Peter Pivonka[1], J Paige Little[1] and Syn Schmitt[1,2,3*]

[1]School of Mechanical, Medical and Process Engineering, Queensland University of Technology, Brisbane, Australia.
[2*]Institute for Modelling and Simulation of Biomechanical Systems, University of Stuttgart, Germany.
[3]Stuttgart Center for Simulation Science (SC SimTech), University of Stuttgart, Germany.

*Corresponding author(s). E-mail(s): schmitt@simtech.uni-stuttgart.de;

Contents

1 Generic muscle parameters **2**

2 Muscle-specific parameters **3**

3 Model-specific structural resolution of internal forces **7**

4 Model-specific evaluation of the FSU stiffness **8**

5 Ligament forces and strain **9**

List of Tables

1 Generic muscle parameters 2
2 Muscle-specific parameters of abdominal muscles 3
3 Muscle-specific parameters of back muscles 4

1 Generic muscle parameters

Table 1: Generic muscle parameters

CE	activation dynamics	m [s^{-1}] 15.0*	ϖ [ ] 8.0	ν [ ] 3.0	q_0 [ ] 0.001
	force-length relation	ΔW_{asc} [ ] 0.35	$\nu_{\text{CE,asc}}$ [ ] 3.0	ΔW_{des} [ ] 0.35*	$\nu_{\text{CE,des}}$ [ ] 1.5*
	force-velocity relation	$A_{\text{rel},0}$ [ ] 0.2	$B_{\text{rel},0}$ [s^{-1}] 2.0	$\mathcal{F}_{\text{max,ecc}}$ [ ] 1.8*	$\mathcal{S}_{\text{ecc}}$ [ ] 2.0*
PEE		$\mathcal{L}_{\text{PEE},0}$ [ ] 0.95	ν_{PEE} [ ] 4.0	$\mathcal{F}_{\text{max,PEE}}$ [ ] 0.2	
SEE		$\Delta U_{\text{SEE,nll}}$ [ ] 0.06	$\Delta U_{\text{SEE,l}}$ [ ] 0.02	$\Delta F_{\text{SEE},0}$ [N] $F_{\text{max}}/3$	
SDE		D_{SDE} [ ] 0.3	R_{SDE} [ ] 0.01		

Generic muscle parameters as used in our recently published model (Mörl et al., 2020). Note, that parameter values with an asterisk were inadvertently misstated in Table 5 of Mörl et al. (2020) and corrected here using the values from Mörl et al. (2012). A comprehensive parameter description can be found in Günther et al. (2007) and Haeufle et al. (2014). Parameters for the activation dynamics of the contractile element (CE) (Hatze, 1977) are implemented according to Rockenfeller et al. (2015) in its recently revised form (Rockenfeller and Günther, 2018)

2 Muscle-specific parameters

Table 2: Muscle-specific parameters of abdominal muscles

Muscle name	PCSA[cm^2]	F_{max} [N]	m_{ratio} [ ]
Rectus abdominis		[C]/4	[C]
M_rectus_abdominis_1	1.42	32.60	0.788
M_rectus_abdominis_2	1.42	32.60	0.788
M_rectus_abdominis_3	1.42	32.60	0.788
M_rectus_abdominis_4	1.42	32.60	0.788
Externus oblique		[C]	[C]
M_externus_oblique_1	1.96	45.080	0.389
M_externus_oblique_2	2.32	53.360	0.410
M_externus_oblique_3	2.43	55.890	0.455
M_externus_oblique_4	2.34	53.820	0.470
M_externus_oblique_5	2.73	62.790	0.480
M_externus_oblique_6	3.97	91.310	0.50
Internus oblique		[C]	[C]
M_internus_oblique_1	1.85	42.550	0.40
M_internus_oblique_2	2.24	51.520	0.40
M_internus_oblique_3	2.26	51.980	0.40
M_internus_oblique_4	2.68	61.640	0.60
M_internus_oblique_5	2.35	54.050	0.60
M_internus_oblique_6	2.07	47.610	0.60
Psoas major		[C]	[C]
M_psoas_major_L1L2Pelvis_IVD	1.20	27.600	0.800
M_psoas_major_L1Pelvis_TP	0.61	14.030	0.800
M_psoas_major_L1Pelvis_VB	2.11	48.530	0.800
M_psoas_major_L2L3Pelvis_IVD	1.19	27.370	0.800
M_psoas_major_L2Pelvis_TP	2.11	48.530	0.800
M_psoas_major_L3L4Pelvis_IVD	0.36	8.280	0.800
M_psoas_major_L3Pelvis_TP	1.01	23.230	0.800
M_psoas_major_L4L5Pelvis_IVD	0.79	18.170	0.800
M_psoas_major_L4Pelvis_TP	1.61	37.030	0.800
M_psoas_major_L5Pelvis_TP	1.73	39.790	0.800
M_psoas_major_L5Pelvis_VB	1.91	43.930	0.800

Muscle-specific parameters (physiological cross-sectional area PCSA, ratio of the muscle fibre length to the length of the muscle tendon unit m_{ratio}) were taken from literature (Key: [C]: Christophy et al. (2012)) and used to derive the three modelling parameters: Maximum isometric force F_{max}, optimal fibre length $l_{CE,opt}$ and tendon slack length $l_{SEE,0}$. For the calculation of F_{max} we assumed a maximum isometric stress of $\sigma = 23$ N/cm^2

Table 3: Muscle-specific parameters of back muscles

Muscle name	PCSA[cm^2]	$F_{\max}$ [N]	m_{ratio} [ ]
Erectus spinae	[C]		[C]
M_longissimus_thoracis_pars_lumborum_L1S1	0.79	18.170	0.419
M_longissimus_thoracis_pars_lumborum_L2S1	0.91	20.930	0.433
M_longissimus_thoracis_pars_lumborum_L3S1	1.03	23.690	0.436
M_longissimus_thoracis_pars_lumborum_L4S1	1.10	25.300	0.438
M_longissimus_thoracis_pars_lumborum_L5S1	1.16	26.680	0.440
M_longissimus_thoracis_pars_thoracis_R4L4	0.23	5.290	0.330
M_longissimus_thoracis_pars_thoracis_R5L5	0.22	5.060	0.330
M_longissimus_thoracis_pars_thoracis_R6Pelvis	0.32	7.360	0.353
M_longissimus_thoracis_pars_thoracis_R7Pelvis	0.39	8.970	0.333
M_longissimus_thoracis_pars_thoracis_R8Pelvis	0.63	14.490	0.290
M_longissimus_thoracis_pars_thoracis_R9Pelvis	0.73	16.790	0.254
M_longissimus_thoracis_pars_thoracis_R10Pelvis	0.80	18.400	0.327
M_longissimus_thoracis_pars_thoracis_R11Pelvis	0.84	19.320	0.370
M_longissimus_thoracis_pars_thoracis_R12Pelvis	0.69	15.870	0.300
M_longissimus_thoracis_pars_thoracis_T1L1	0.29	6.670	0.260
M_longissimus_thoracis_pars_thoracis_T2L2	0.57	13.110	0.257
M_longissimus_thoracis_pars_thoracis_T3L3	0.56	12.880	0.257
M_longissimus_thoracis_pars_thoracis_T4L4	0.23	5.290	0.257
M_longissimus_thoracis_pars_thoracis_T5L5	0.22	5.060	0.257
M_longissimus_thoracis_pars_thoracis_T6Pelvis	0.32	7.360	0.267
M_longissimus_thoracis_pars_thoracis_T7Pelvis	0.39	8.970	0.306
M_longissimus_thoracis_pars_thoracis_T8Pelvis	0.63	14.490	0.346
M_longissimus_thoracis_pars_thoracis_T9Pelvis	0.73	16.790	0.330
M_longissimus_thoracis_pars_thoracis_T10Pelvis	0.80	18.400	0.330
M_longissimus_thoracis_pars_thoracis_T11Pelvis	0.84	19.320	0.330
M_longissimus_thoracis_pars_thoracis_T12Pelvis	0.69	15.870	0.330
M_iliocostalis_lumborum_pars_lumborum_L1S1	1.08	24.840	0.274
M_iliocostalis_lumborum_pars_lumborum_L2S1	1.54	35.420	0.274
M_iliocostalis_lumborum_pars_lumborum_L3S1	1.82	41.860	0.274
M_iliocostalis_lumborum_pars_lumborum_L4S1	1.89	43.470	0.274
M_iliocostalis_lumborum_pars_thoracis_R10Pelvis	1.00	23.000	0.600
M_iliocostalis_lumborum_pars_thoracis_R11Pelvis	1.23	28.290	0.640
M_iliocostalis_lumborum_pars_thoracis_R12Pelvis	1.47	33.810	0.640
M_iliocostalis_lumborum_pars_thoracis_R5Pelvis	0.23	5.290	0.381
M_iliocostalis_lumborum_pars_thoracis_R6Pelvis	0.31	7.130	0.417
M_iliocostalis_lumborum_pars_thoracis_R7Pelvis	0.39	8.970	0.452
M_iliocostalis_lumborum_pars_thoracis_R8Pelvis	0.34	7.820	0.462
M_iliocostalis_lumborum_pars_thoracis_R9Pelvis	0.50	11.500	0.600
Intertransversarii		[M]	[M]
M_intertransversarii_mediales_L1L2	-	21.420	0.500
M_intertransversarii_mediales_L2L3	-	21.420	0.500
M_intertransversarii_mediales_L3L4	-	21.420	0.500
M_intertransversarii_mediales_L4L5	-	21.420	0.500
Multifidus	[C]		[C]
M_multifidus_multifascicle_L1L5	0.42	9.660	0.730
M_multifidus_multifascicle_L1S1	0.36	8.280	0.730
M_multifidus_multifascicle_L1S1caudal	0.60	13.800	0.730
M_multifidus_multifascicle_L2S1	0.50	11.390	0.727
M_multifidus_multifascicle_L2S1caudal	0.50	11.390	0.727
M_multifidus_multifascicle_L2S1cranial	0.39	8.970	0.727
M_multifidus_multifascicle_L3S1	0.52	11.960	0.709
M_multifidus_multifascicle_L3S1caudal	0.52	11.960	0.709
M_multifidus_multifascicle_L3S1cranial	0.52	11.960	0.709
M_multifidus_multifascicle_L4S1	0.47	10.810	0.667
M_multifidus_multifascicle_L4S1caudal	0.47	10.810	0.667
M_multifidus_multifascicle_L4S1cranial	0.47	10.810	0.667
M_multifidus_multifascicle_L5S1	0.23	5.290	0.667
M_multifidus_multifascicle_L5S1caudal	0.23	5.290	0.667

M_multifidus_multifascicle_L5S1cranial	0.23	5.290	0.667
M_multifidus_laminarfibres_L1L3	0.19	4.370	0.681
M_multifidus_laminarfibres_L2L4	0.22	5.060	0.681
M_multifidus_laminarfibres_L3L5	0.23	5.290	0.681
M_multifidus_laminarfibres_L4S1	0.17	3.910	0.681
M_multifidus_laminarfibres_L5S1	0.36	8.280	0.681
M_multifidus_singlefascicle_L1L4	0.40	9.200	0.661
M_multifidus_singlefascicle_L2L5	0.39	8.970	0.677
M_multifidus_singlefascicle_L3S1	0.54	12.420	0.661
M_multifidus_singlefascicle_L4S1	0.47	10.810	0.562
M_multifidus_singlefascicle_L5S1	0.23	5.290	0.562
	[S]		av.[Ba]
M_multifidus_laminarfibres_T1T3	0.40	9.200	0.797
M_multifidus_laminarfibres_T2T4	0.40	9.200	0.797
M_multifidus_laminarfibres_T3T5	0.40	9.200	0.797
M_multifidus_laminarfibres_T4T6	0.40	9.200	0.797
M_multifidus_laminarfibres_T5T7	0.40	9.200	0.797
M_multifidus_laminarfibres_T6T8	0.40	9.200	0.797
M_multifidus_laminarfibres_T7T9	0.40	9.200	0.797
M_multifidus_laminarfibres_T8T10	0.40	9.200	0.797
M_multifidus_laminarfibres_T9T11	0.40	9.200	0.797
M_multifidus_laminarfibres_T10T12	0.40	9.200	0.797
M_multifidus_laminarfibres_T11L1	0.40	9.200	0.797
M_multifidus_laminarfibres_T12L2	0.40	9.200	0.797
M_multifidus_singlefascicle_T1T4	0.40	9.200	0.797
M_multifidus_singlefascicle_T2T5	0.40	9.200	0.797
M_multifidus_singlefascicle_T3T6	0.40	9.200	0.797
M_multifidus_singlefascicle_T4T7	0.40	9.200	0.797
M_multifidus_singlefascicle_T5T8	0.40	9.200	0.797
M_multifidus_singlefascicle_T6T9	0.40	9.200	0.797
M_multifidus_singlefascicle_T7T10	0.40	9.200	0.797
M_multifidus_singlefascicle_T8T11	0.40	9.200	0.797
M_multifidus_singlefascicle_T9T12	0.40	9.200	0.797
M_multifidus_singlefascicle_T10L1	0.40	9.200	0.797
M_multifidus_singlefascicle_T11L2	0.40	9.200	0.797
M_multifidus_singlefascicle_T12L3	0.40	9.200	0.797
Semispinalis	[Ba]		av.[Ba]
M_semispinalis_T1T8_s	0.160	3.680	0.597
M_semispinalis_T2T9_s	0.160	3.680	0.597
M_semispinalis_T3T10_s	0.160	3.680	0.597
M_semispinalis_T4T11_s	0.160	3.680	0.597
M_semispinalis_T5T12_s	0.160	3.680	0.597
Interspinalis		[M]	[M]
M_interspinalis_L1L2	-	13.430	0.500
M_interspinalis_L2L3	-	13.430	0.500
M_interspinalis_L3L4	-	13.430	0.500
M_interspinalis_L4L5	-	13.430	0.500
	est.[Br]		est.[CD]
M_interspinalis_T10T11	0.50	11.500	0.530
M_interspinalis_T11T12	0.50	11.500	0.530
M_interspinalis_T12L1	0.50	11.500	0.530
M_interspinalis_T1T2	0.50	11.500	0.530
M_interspinalis_T2T3	0.50	11.500	0.530
M_interspinalis_T3T4	0.50	11.500	0.530
M_interspinalis_T4T5	0.50	11.500	0.530
M_interspinalis_T5T6	0.50	11.500	0.530
M_interspinalis_T6T7	0.50	11.500	0.530
M_interspinalis_T7T8	0.50	11.500	0.530
M_interspinalis_T8T9	0.50	11.500	0.530
M_interspinalis_T9T10	0.50	11.500	0.530

Spinalis	[D]		[D]
M_spinalis_T1L5	0.20	4.600	0.259
M_spinalis_T2L4	0.20	4.600	0.259
M_spinalis_T3L3	0.20	4.600	0.259
M_spinalis_T4L2	0.20	4.600	0.259
M_spinalis_T5L1	0.20	4.600	0.259
M_spinalis_T6T12	0.20	4.600	0.259
M_spinalis_T7T11	0.20	4.600	0.259
M_spinalis_T8T10	0.20	4.600	0.259

Muscle-specific parameters (physiological cross-sectional area PCSA, ratio of the muscle fibre length to the length of the muscle tendon unit m_{ratio}) were taken or estimated from literature and used to derive the three modelling parameters: Maximum isometric force F_{max}, optimal fibre length $l_{\mathrm{CE,opt}}$ and tendon slack length $l_{\mathrm{SEE,0}}$. For the calculation of F_{max} we assumed a maximum isometric stress of $\sigma = 23$ N/cm^2. The source of this data is given at the top of each muscle column. Key: [C]: Christophy et al. (2012), [M]: Mörl et al. (2020), [S]: Stokes and Gardner-Morse (1999), [D]: Delp et al. (2001), [Br]: Brolin et al. (2005), [Ba]: Bayoglu et al. (2017), [CD]: Cramer and Darby (2005)

3 Model-specific structural resolution of internal forces

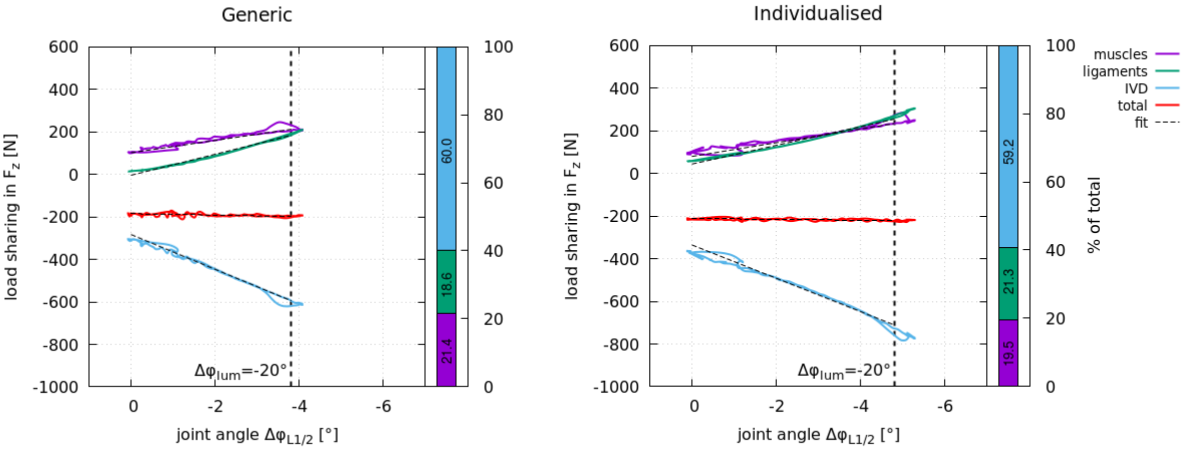

(a)

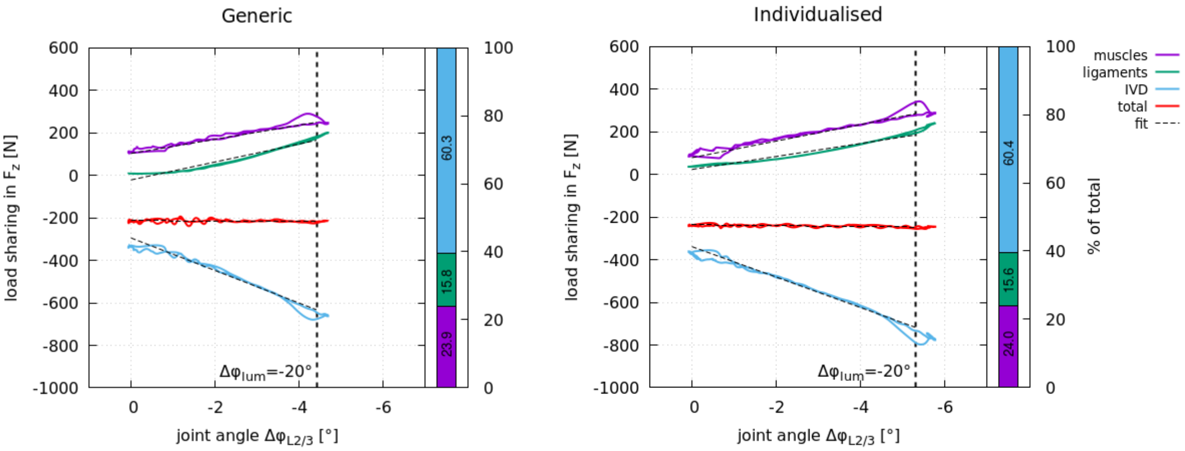

(b)

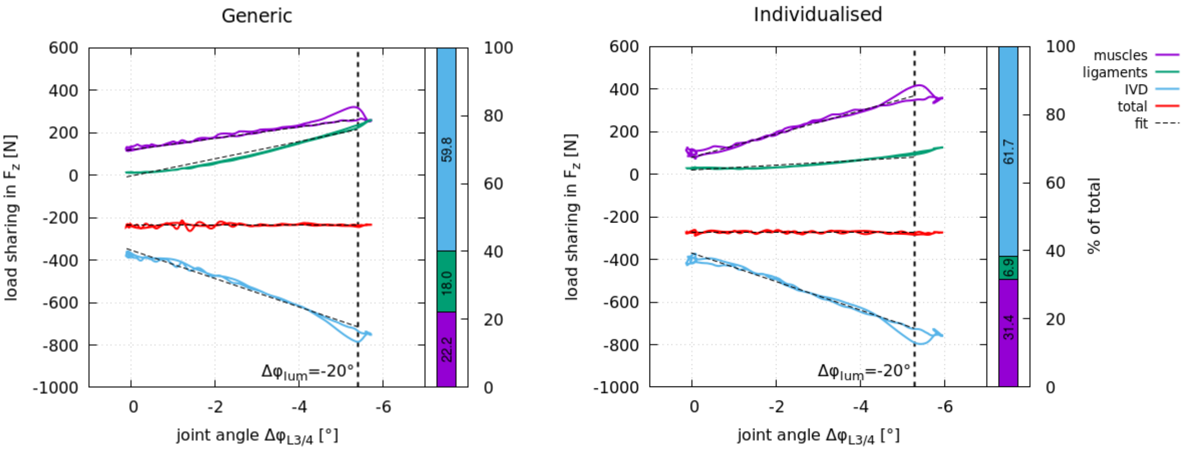

(c)

4 Model-specific evaluation of the FSU stiffness

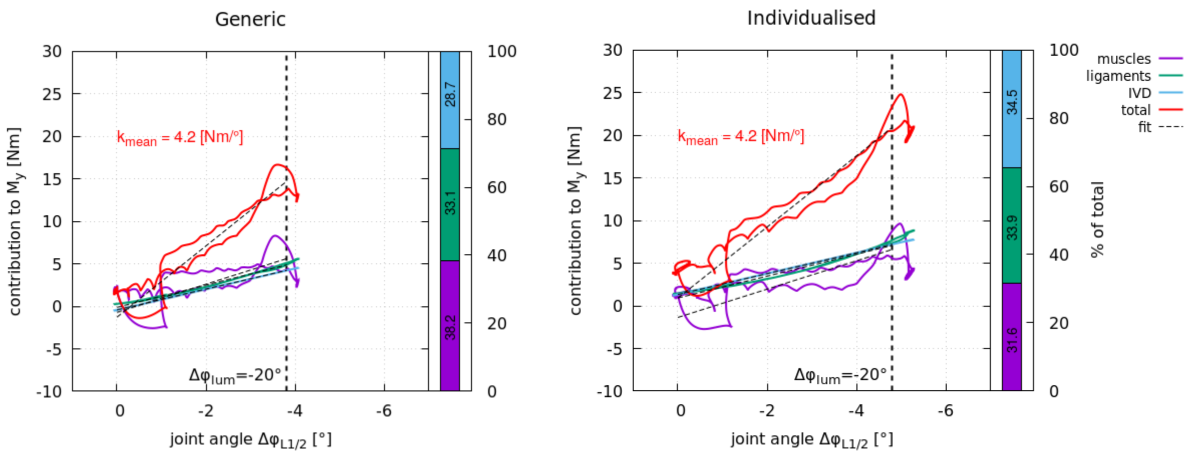

(a)

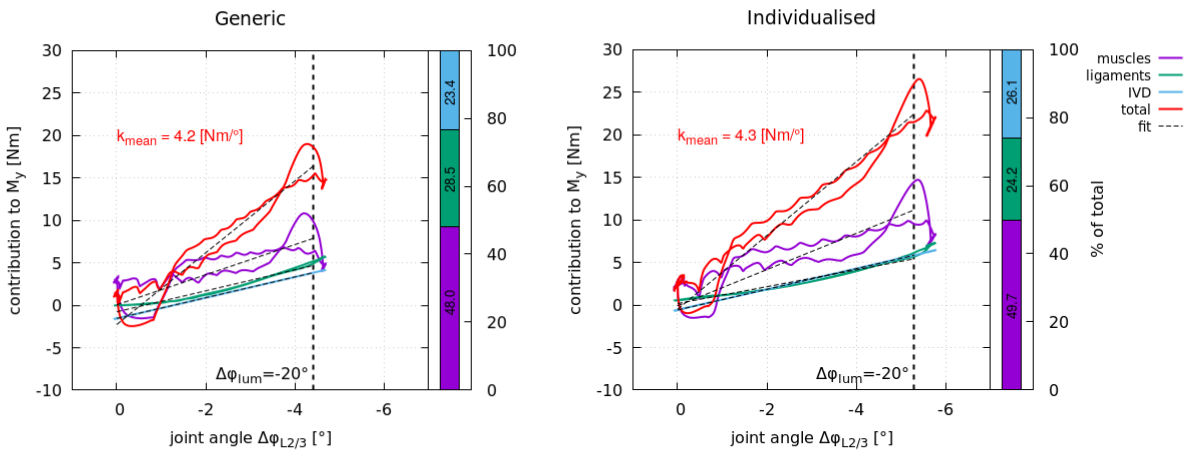

(b)

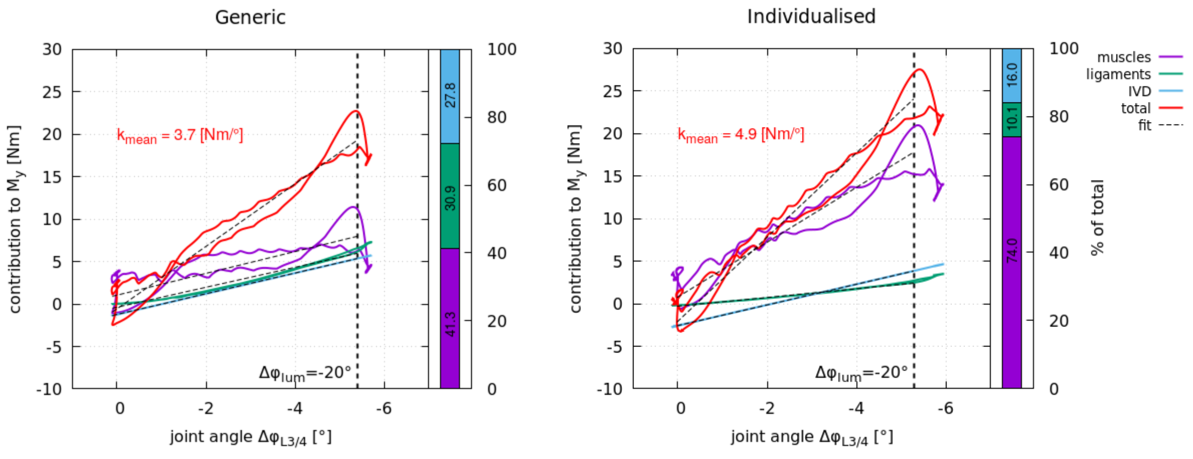

(c)

5 Ligament forces and strain

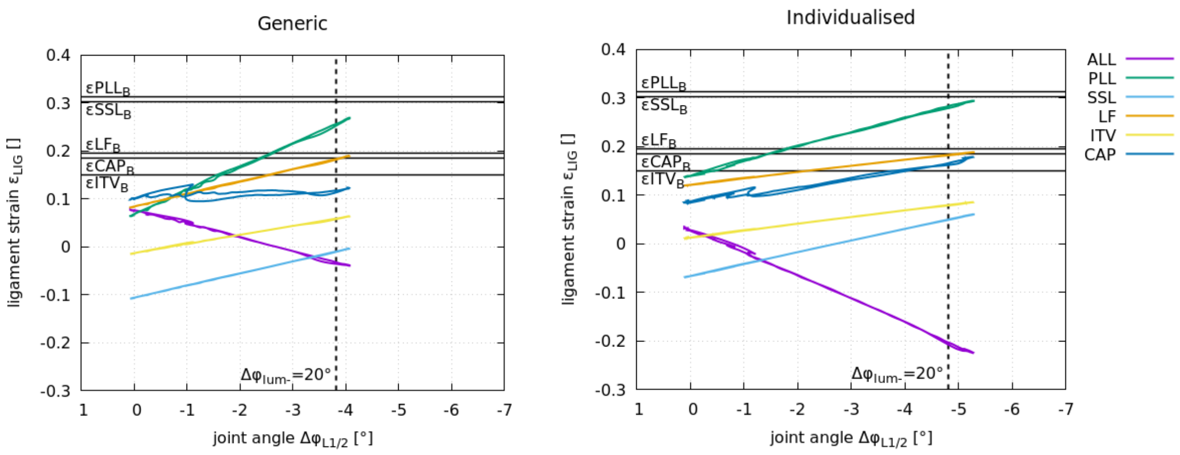

(a)

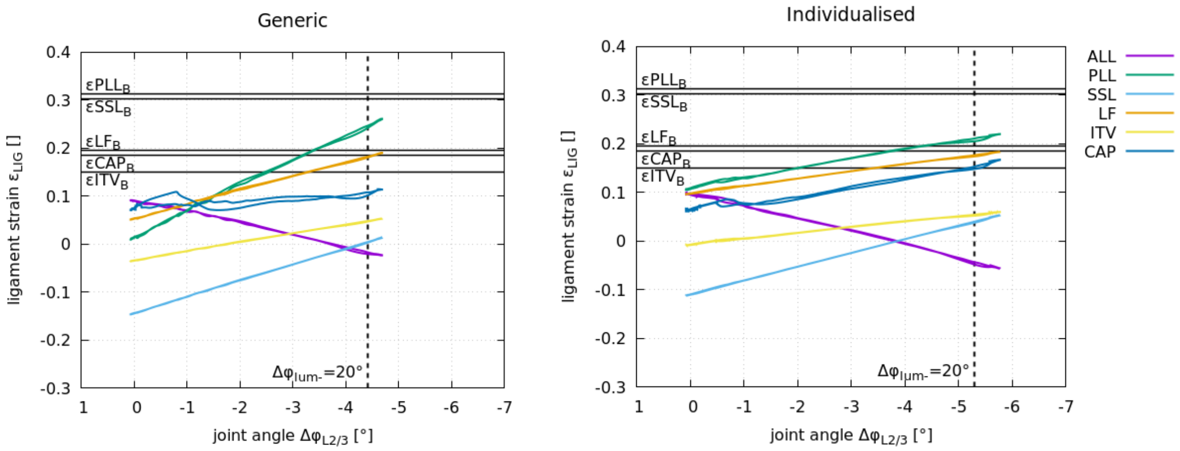

(b)

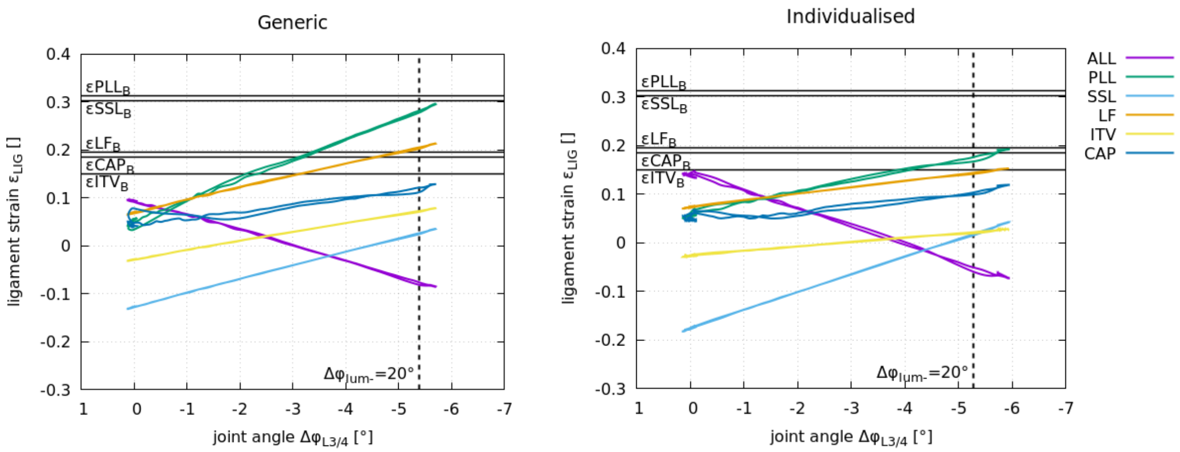

(c)

References

Bayoglu R, Geeraedts L, Groenen KH, et al. (2017) Twente spine model: A complete and coherent dataset for musculo-skeletal modeling of the thoracic and cervical regions of the human spine. Journal of Biomechanics 58:52–63. https://doi.org/10.1016/j.jbiomech.2017.04.003

Brolin K, Halldin P, Leijonhufvud I (2005) The effect of muscle activation on neck response. Traffic Injury Prevention 6(1):67–76. https://doi.org/10.1080/15389580590903203

Christophy M, Faruk Senan NA, Lotz JC, et al. (2012) A musculoskeletal model for the lumbar spine. Biomechanics and modeling in mechanobiology 11(1-2):19–34. https://doi.org/10.1007/s10237-011-0290-6

Cramer GD, Darby SA (2005) Basic and clinical anatomy of the spine, spinal cord, and ANS, 2nd edn. Elsevier Mosby, St. Louis, Missouri

Delp SL, Suryanarayanan S, Murray WM, et al. (2001) Architecture of the rectus abdominis, quadratus lumborum, and erector spinae. Journal of Biomechanics 34(3):371–375. https://doi.org/10.1016/S0021-9290(00)00202-5

Günther M, Schmitt S, Wank V (2007) High-frequency oscillations as a consequence of neglected serial damping in Hill-type muscle models. Biological Cybernetics 97(1):63–79. https://doi.org/10.1007/s00422-007-0160-6

Haeufle D, Günther M, Bayer A, et al. (2014) Hill-type muscle model with serial damping and eccentric force-velocity relation. Journal of Biomechanics 47(6):1531–1536. https://doi.org/10.1016/j.jbiomech.2014.02.009

Hatze H (1977) A myocybernetic control model of skeletal muscle. Biological cybernetics 25(2):103–119. https://doi.org/10.1007/BF00337268

Mörl F, Siebert T, Schmitt S, et al. (2012) Electro-mechanical delay in hill-type muscle models. Journal of Mechanics in Medicine and Biology 12(05):1250,085. https://doi.org/10.1142/S0219519412500856

Mörl F, Günther M, Riede JM, et al. (2020) Loads distributed in vivo among vertebrae, muscles, spinal ligaments, and intervertebral discs in a passively flexed lumbar spine. Biomechanics and Modeling in Mechanobiology 19(6):2015–2047. https://doi.org/10.1007/s10237-020-01322-7

Rockenfeller R, Günther M (2018) Inter-filament spacing mediates calcium binding to troponin: A simple geometric-mechanistic model explains the shift of force-length maxima with muscle activation. Journal of Theoretical Biology 454:240–252. https://doi.org/10.1016/j.jtbi.2018.06.009

Rockenfeller R, Günther M, Schmitt S, et al. (2015) Comparative sensitivity analysis of muscle activation dynamics. Computational and mathematical methods in medicine 2015(4):1–16. https://doi.org/10.1155/2015/585409

Stokes IA, Gardner-Morse M (1999) Quantitative anatomy of the lumbar musculature. Journal of Biomechanics 32(3):311–316. https://doi.org/10.1016/S0021-9290(98)00164-X
